# Cutaneous wound healing in aged, high fat diet-induced obese female or male C57BL/6 mice

**DOI:** 10.18632/aging.103064

**Published:** 2020-04-15

**Authors:** Marta Kopcewicz, Katarzyna Walendzik, Joanna Bukowska, Anna Kur-Piotrowska, Sylwia Machcinska, Jeffrey M. Gimble, Barbara Gawronska-Kozak

**Affiliations:** 1 Institute of Animal Reproduction and Food Research, Polish Academy of Sciences, Olsztyn, Poland; 2 LaCell LLC, New Orleans, LA 70112, USA; 3 Obatala Sciences Inc., New Orleans, LA 70148, USA; 4 Departments of Medicine, Structural and Cellular Biology, and Surgery and Center for Stem Cell Research and Regenerative Medicine, Tulane University School of Medicine, New Orleans, LA 70112, USA

**Keywords:** age, sex, diet, skin, wound

## Abstract

Since there are limited studies analyzing the impact of age, sex and obesity on cutaneous repair, the current study evaluated excisional skin wound healing as a function of age, sex and diet in C57BL/6 mice subjected to either low (LFD) or high (HFD) fat diet. Older mice accumulated increased body fat relative to younger mice under HFD. Skin wound healing at particular stages was affected by age in the aspect of Tgfβ-1, MCP-1, Mmp-9 and Mmp-13 expression. The most profound, cumulative effect was observed for the combination of two parameters: age and sex. While skin of younger males displayed extremely high collagen 1 and collagen 3 expression, younger females showed exceptionally high Mmp-13 expression at day 3 and 7 after injury. Diet as a single variable modified the thickness of dermis due to increased dermal White Adipose Tissue (dWAT) accumulation in mice fed HFD. The combination of age and diet affected the re-epithelialization and inflammatory response of injured skin. Overall, our data indicate that age has the most fundamental impact although all components (age, sex and diet) contribute to skin repair.

## INTRODUCTION

In adult mammals, skin wound healing is a complex repair process which leads to rapid and effective wound closure. Cutaneous wound healing encompasses a sequence of overlapping phases, including inflammation, new tissue formation and tissue remodelling [1, 2]. The disruption of blood vessels during the skin injury causes blood and fluid loss. The formation of fibrin clot, which provides a temporary scaffold for migrating cells, re-establishes skin hemostasis [1, 2]. In the inflammatory phase, platelet aggregation is followed by the recruitment of polymorphonuclear leukocytes (neutrophils) and invasion of blood monocytes which subsequently differentiate into macrophages [3]. The components of the inflammatory response remove debris and prevent infection. During the second stage of wound healing, the new tissue formation, different cell types are involved in order to achieve permanent closure of the wound gap and restore the protective barrier skin function. Keratinocytes proliferate and migrate over the injured dermis in the process of re-epithelialization, new blood vessels are formed and extracellular matrix is synthesized by interacting fibroblasts and myofibroblasts. Remodelling is the final stage of the skin wound healing process which may last for a year or longer. This phase serves to restore the regular architecture of the dermis following injury and reorganizes the immature extracellular matrix by re-balancing the dynamic between collagen synthesis, arrangement and degradation [4]. This remodelling phase involves apoptosis of a variety of cell types within the wound site. The scar tissue that is formed in post injured area never achieves the strength and functionality of uninjured skin [2].

Multiple factors influencing cutaneous injury repair can lead to improper or impaired wound healing. Factors that influence skin wound healing can be categorized as local, such as oxygenation and infection, or systemic [5], such as obesity, age and sex. Obesity is defined as excessive fat accumulation, which constitutes a risk for human health (World Health Organization (WHO); https://www.who.int/topics/obesity/en/). In 2016 nearly half a billion adults worldwide were obese (Global Health Observatory (GHO); https://www.who.int/gho/ncd/risk_factors/overweight_text/en/). Over the past 20 years, adult and childhood obesity rates have doubled, creating a risk of heart disease, hypertension, cancer, stroke and diabetes. Obesity alters the barrier function of the skin, causing increased transepidermal water loss [6]. Moreover, obesity predispose individuals to the occurrence of impaired wound healing promoting pressure and venous ulcers [5]. Several studies indicate that the increment of adipose tissue may contribute to impaired dermal functions and defects in skin wound healing [7, 8]. Furthermore, adipocytes that are the source of multiple bioactive substances, known as adipose-derived secreted factors (adipokines), may exert pro-inflammatory or anti-inflammatory effects. Imbalanced/disrupted expression of these adipokines secondary to adipose tissue dysfunction, can be linked to a chronic low-grade inflammatory state and injury repair complications [9].

Old age is one of the main systemic factors affecting skin wound healing process. The WHO estimated that between year 2015 and 2050 the number of people aged 60 years and older will rise from 900 million to 2 billion (WHO; https://www.who.int/features/factfiles/ageing/en/). The skin’s structure changes with advancing age due to the sum of extrinsic and intrinsic influences. Extrinsic modulators reflect the cumulative effect of environmental insults, such as UV radiation, whereas intrinsic aging relates to skin changes that are independent of environmental factors, such as genetic influences. Advancing age is accompanied by decreases in dermal cellular content, blood flow and lymphatic drainage [10], collagen content, and elasticity [11]. All these age-related changes lead to alterations in skin wound healing. Multiple clinical studies have shown that skin wound healing in healthy elderly individuals is delayed, although it is not impaired in terms of the quality of healing [10, 12]. The most commonly described modifications of cutaneous injury repair in the elderly are delayed infiltration of T-cells into the wound with reduced macrophages phagocytic capacity, [13, 14], delayed re-epithelialization and angiogenesis [14], altered expression of growth factors and their receptors, such as platelet derived growth factor (PDGF) and epidermal growth factor (EGF) [15] as well as an imbalance in the Mmp/Timp-1 (matrix metalloproteinases/ tissue inhibitor of matrix metalloproteinases 1) ratio with increased levels of Mmps [16] and decreased Timp-1 expression [17]. An additional age-related modification noted in murine incisional skin wound healing has been a change in the immunolocalization of transforming growth factor beta (Tgfβ) isoforms [11].

Accumulating evidence indicates that sexual dimorphism exists in the skin structure and skin wound healing process. In human, men’s skin is thicker than women’s at all ages [18]. Skin thickness decreases linearly with advancing age, starting at the age of 50 in women and at the age of 20 in men [19]. Likewise, murine studies have shown that male mice have thicker dermis but thinner epidermis and hypodermis than females [20, 21].

While the ability of individual biological factor, such as age or obesity, to disrupt subsequent phases of wound repair have been described, comprehensive analyzes examining the combined effects of age, sex and obesity on cutaneous wound healing are limited. Hence, the present study was designed to explore the effect of age, sex and LFD vs HFD on skin wound healing in C57BL/6J (B6) mice.

## RESULTS

**Figure 1:**
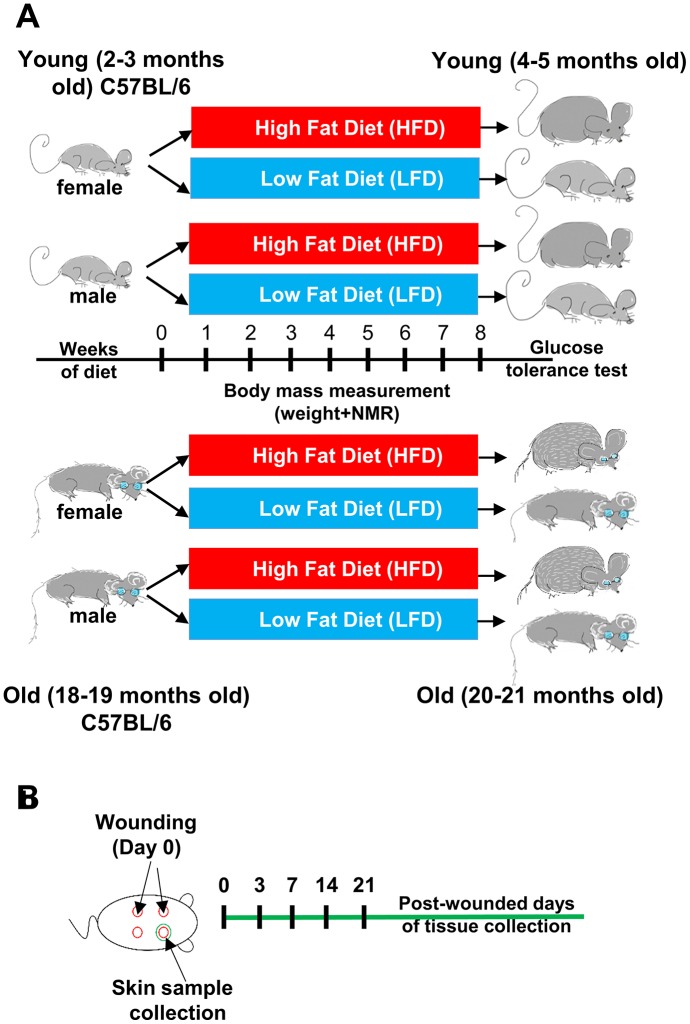
**Scheme of the experimental design.** (**A**) Young (2-3 months old) female (n=48) and male (n=48), and old (18-19 months old) female (n=48) and male (n=48) C57BL/6 (B6) mice were fed for 8 weeks on either LFD or HFD. (**B**) Mice were injured at day 0. Skin tissues were collected at day 0 (uninjured control) and post-wounded days: 3, 7, 14 and 21, n=4-6 mice per time point/per group.

Young (2-3 months) and old (18-19 months) B6 mice of both sexes were assigned into groups fed for a period of 8 weeks with either chow diet (low fat diet- LFD 13 kcal% fat) or high fat diet (HFD 59 kcal% fat; Figure 1A). After 8 weeks of HFD or LFD, 4-5 months (young) and 20-21 months (old) cohorts underwent 4x4mm diameter skin punches. Skin tissues samples were collected subsequently at day 0 and 3, 7, 14 and 21 after wounding (Figure 1B).

### Body composition

**Figure 2:**
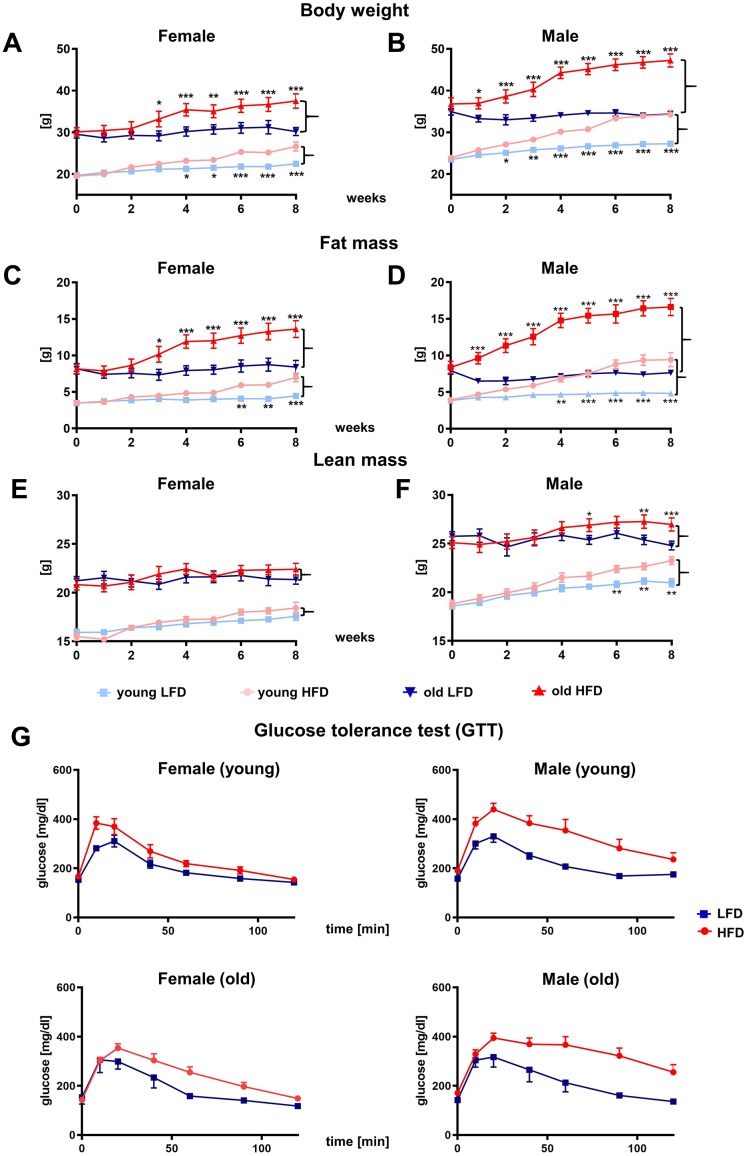
Effect of HFD *vs.* LFD on body weight (**A**, **B**), fat mass (**C**, **D**), lean mass (**E**, **F**) and glucose tolerance test (**G**) of B6 female (**A**, **C**, **E**, **G**) and male (**B**, **D**, **F**, **G**) mice. Body weight and body composition analyzed by nuclear magnetic resonance (NMR), were measured weekly during 8-week feeding study (n=192 total mice including: n=96 per LFD and n=96 per HFD). Data are the lsmean ±SE, asterisks indicate significant differences between animals fed HFD vs LFD *p<0.05, **p<0.01, ***p<0.001.

Over the course of the 8 week feeding experiment, an increase in body weight was stimulated by HFD in both males and females (Figure 2A, 2B; Supplementary Table 1). The most evident differences in body weight gain was detected between females (Figure 2A) and males (Figure 2B; Supplementary Table 2). The stable, linear increase in body weight was substantially greater for males relative to females, regardless of age.

The difference in body weight gain between HFD and LFD reached statistical significance within 2 weeks of diet (p<0.05) for young males and 1 week for old (p<0.05), whereas for females this was achieved after 4 weeks of diet for young (p<0.05) and 3 weeks for old (p<0.05). The different response between female and male mice to HFD indicates a clear role of sex to dietary regiment (Supplementary Tables 1–3).

Weekly body composition analyzes performed by nuclear magnetic resonance (NMR) confirmed sex differences in body weight gain (Figure 2C–2F; Supplementary Table 4–9). The increase in body weight in males and females fed HFD was achieved primarily through fat mass accumulation (Figure 2C, 2D). The statistically significant differences in body fat mass gain between old HFD vs LFD male mice were apparent after 1 week of diet (p<0.001) and continued to increase until the end of dietary program (p<0.001), whereas for old females it was delayed until the 3^rd^ week (p<0.05, Figure 2C, 2D; Supplementary Table 4–6). No differences in body fat content was observed in mice fed LFD.

The lean mass content did not change substantially during the 8 weeks of feeding study in the young or old females (Figure 2E). Interestingly, for males fed HFD, the lean mass content increased (Figure 2F) indicating that both fat and lean mass contributed to overall body weight gains (Figure 2B; Supplementary Table 7–9). The glucose tolerance test (GTT) performed at the end of the 8^th^ week showed impaired glucose tolerance for HFD mice which was most severe for males (Figure 2G).

### Histological analysis of the skin at the end of 8^th^ week of the HFD or LFD program.

**Figure 3:**
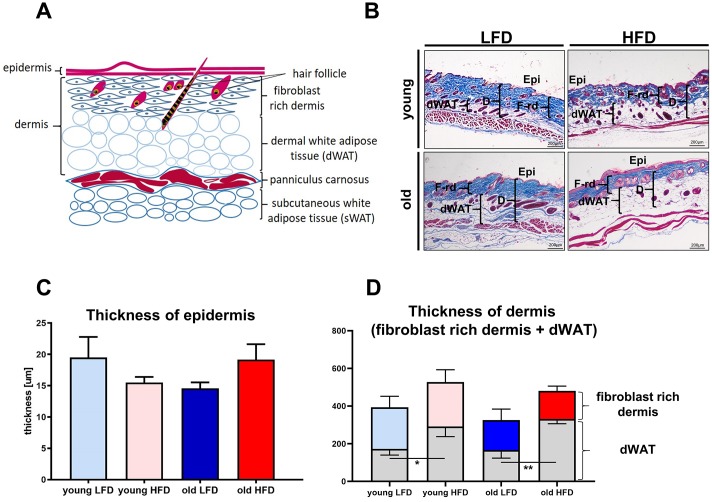
**Histological analysis of skin structure and thickness.** Scheme of skin structure (**A**), histological skin sections stained with Masson trichrome and collected from young or old mice fed for a period of 8 weeks LFD or HFD (**B**), quantification of the skin layers thickness: epidermis (**C**) and dermis (**D**). The measurement of skin thickness were performed on histological slides collected from n=24 mice (n=6 per group). Epi - epidermis, dWAT - dermal white adipose tissue, F-rd fibroblast rich dermis; scale bar 200 μm, The bars indicate lsmean ±SE *p<0.05, **p<0.01.

Mammalian skin is comprised of three structural layers: epidermis, dermis and subcutaneous white adipose tissue (sWAT), which in rodents is separated from dermis by a thin layer of skeletal muscle known as the *panniculus carnosus* (Figure 3A) [22]. Recent studies have further defined the heterogeneity of the dermis [22–24], pointing out its structural division into papillary and reticular dermis, and dermal white adipose tissue (dWAT) [22, 23] (Figure 3A) identified as the layer of adipocytes within the reticular dermis of the skin [25].

To analyze the impact of age and diet on the skin of young and old mice after LFD or HFD, we performed histological measurements of the thickness of the epidermis, the dermis, and the dWAT (Figure 3A). The thickness of epidermis demonstrated no significant differences (Figure 3B–3D) among analyzed groups. The measurement of the total dermis thickness (comprising the fibroblast-rich dermis + dWAT) showed increases in mice fed HFD regardless of age (Figure 3B, 3D, Supplementary Table 10). While measurements of the fibroblast-rich dermis alone decreased in old animals, regardless of diet (Figure 3B, 3D; Supplementary Table 13), the increase in dWAT thickness, as a result of HFD in old mice, compensated for the loss of fibroblast rich dermis (Figure 3B, 3D; Supplementary Tables 11, 12). As a consequence, dermal layer was significantly thicker in HFD relative to LFD old mice and comparable to that observed in young, HFD animals (Figure 3B, 3D).

### Age, sex and obesogenic environment differentially modulate skin wound healing parameters

**Figure 4:**
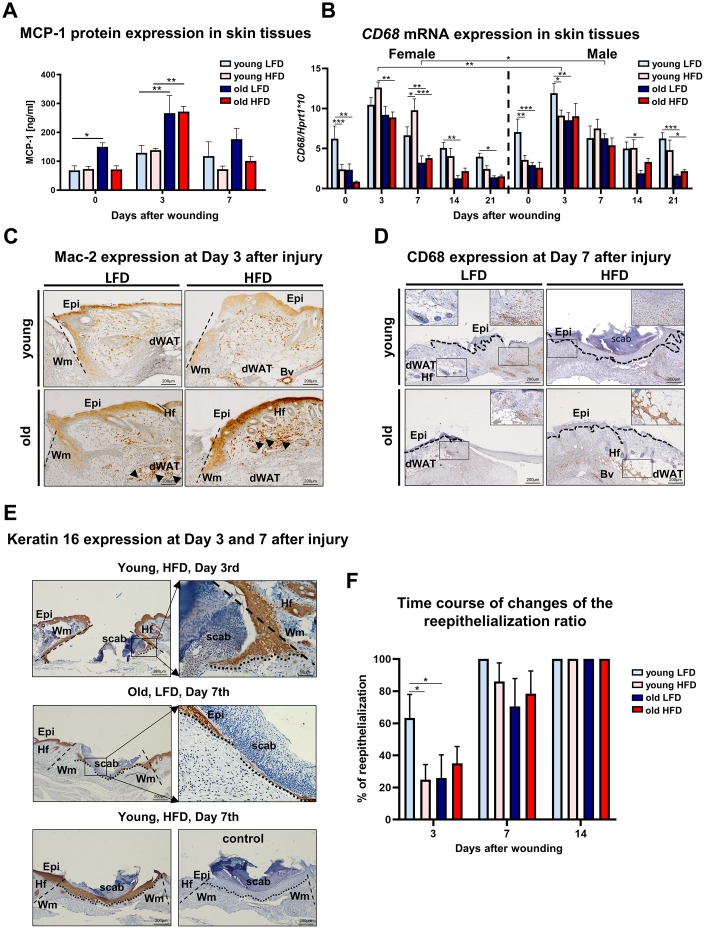
**Inflammatory response and histological analysis of re-epithelialization during skin wound healing.** (**A**) MCP-1 protein levels (n=6 skin samples per group); (**B**) CD68 mRNA expression (n=4-8 skin samples per group); (**C**) Mac-2 and (**D**) CD68 immunohistological localization on skin tissues at post-wounded day 3 (**C**) and day 7 (**D**). Immunohistochemical detection of keratin 16 (**E**) and morphometrical analysis (**F**) of the re-epithelization process in the skin of old, young, LFD or HFD mice (n = 3-5 mice per group). Epi - epidermis, dWAT - dermal white adipose tissue, Wm – wound margin, Hf – hair follicles; control (**E**) of immunohistochemical reaction where the primary antibody were omitted. Histological sections were counterstain with haematoxylin. Scale bar (**C**–**E**) 200μm, insets (**C**–**E**) 50μm. The bars indicate lsmean ±SE *p<0.05, **p<0.01, ***p<0.001.

Skin wound healing occurs in overlapping but distinct stages characterized by inflammation, new tissue formation and remodelling [2]. The inflammatory phase is marked by recruitment to the wound of monocytes which subsequently differentiate into macrophages and together with resident cells (keratinocytes at the wound edge, fibroblasts and endothelial cells) produce MCP-1 (monocytes chemoattractant protein 1), a strongly chemotactic cytokine [26] which further mediates the recruitment of monocytes, mast cells and lymphocytes to the site of injury [27]. To assess the inflammatory phase of skin wound healing in different mice cohorts we measured levels of MCP-1 (Figure 4A), the expression of *CD68* mRNA (Figure 4B) and the immunohistochemical localization of macrophage markers: Mac-2 (galectin-3) (Figure 4C) and CD68 (Figure 4D) in skin tissues. The peak of MCP-1 levels was detected at day 3 after wounding (Figure 4A; Supplementary Table 14). Skin injury in old mice evoked the highest levels of MCP-1 that was not affected by diet (LFD vs HFD) (Figure 4A; Supplementary Table 15). *CD68* mRNA analysis revealed the highest levels of expression at post-wounded day 3 which gradually decreased as the skin wound healing process progressed to day 21, falling to levels comparable to those expressed by uninjured skin (Figure 4B, Supplementary Tables 16–19). Interestingly, in contrast to MCP-1, the highest levels of *CD68* mRNA expression were detected for young mice, regardless sex (Figure 4B, Supplementary Table 19). The effect of age and diet on *CD68* mRNA expression was apparent throughout the skin wound healing process (days 3-21) (Supplementary Table 19). Immunohistological localization of Mac-2 positive cells was identified in the epidermal layer, consistent with reports associating Mac-2 epidermal expression with differentiation and maturation of suprabasal keratinocytes [28] in the wound area, in granulation tissues and in the wound margin (Figure 4C). The accumulation of Mac-2 positive cells was observed in skin sections collected at day 3 (Figure 4C) and, to a lesser extent, day 7 (data not shown) after injury. The most robust infiltration of Mac-2 was observed in old HFD mice (Figure 4C). CD68 immunoreactivity was exclusively detected in the dermal part of the skin after wounding (Figure 4D). Positivity for CD68 was observed among cells spread throughout the post wounded dermis, blood vessels and some adipose tissue (Figure 4D). The strongest CD68 reaction product deposition at post wounded day 7 was observed in dWAT tissues localized at wound margin of old HFD mice (Figure 4D).

New tissue formation involves re-epithelialization, angiogenesis, collagen synthesis and ECM production [5]. The aim of the re-epithelialization process is to cover the wound with the new epithelium. Re-epithelialization involves keratinocyte migration, proliferation and differentiation that is essential for successful cutaneous healing [29, 30]. The analysis of post wounding skin sections stained with: haematoxylin and eosin (H&E) or immunostained for the presence of keratin 16 (Figure 4E), a marker of keratinocytes induction, showed the rate of re-epithelialization measured by percentage of area covered by newly formed epithelium in the wound. The fastest rate of wound re-epithelialization was detected for young, LFD mice: 63.4 % (±11.5) at day 3 and 100% (±19.9) at day 7 (Figure 4F). In the remaining groups of mice, the re-epithelialization rate was significantly slower. At day 3^rd^ after injury, young, HFD mice displayed 24.8% (±10.9), old LFD mice 25.9% (±14.0) and old HFD mice 35% (±12.2) of wound coverage. The combined effect of age and diet on re-epithelialization process was detected at day 3^rd^ after wounding (Supplementary Table 20–22). At the day 7, the percentage of re-epithelialization were 86.2% (±14) for young HFD, 70.5% (±17.2) old LFD and 78.4% (±12.2) old HFD mice. By day 14 wounds of all studied cohorts were fully re-epithelialized (Figure 4E, 4F).

**Figure 5:**
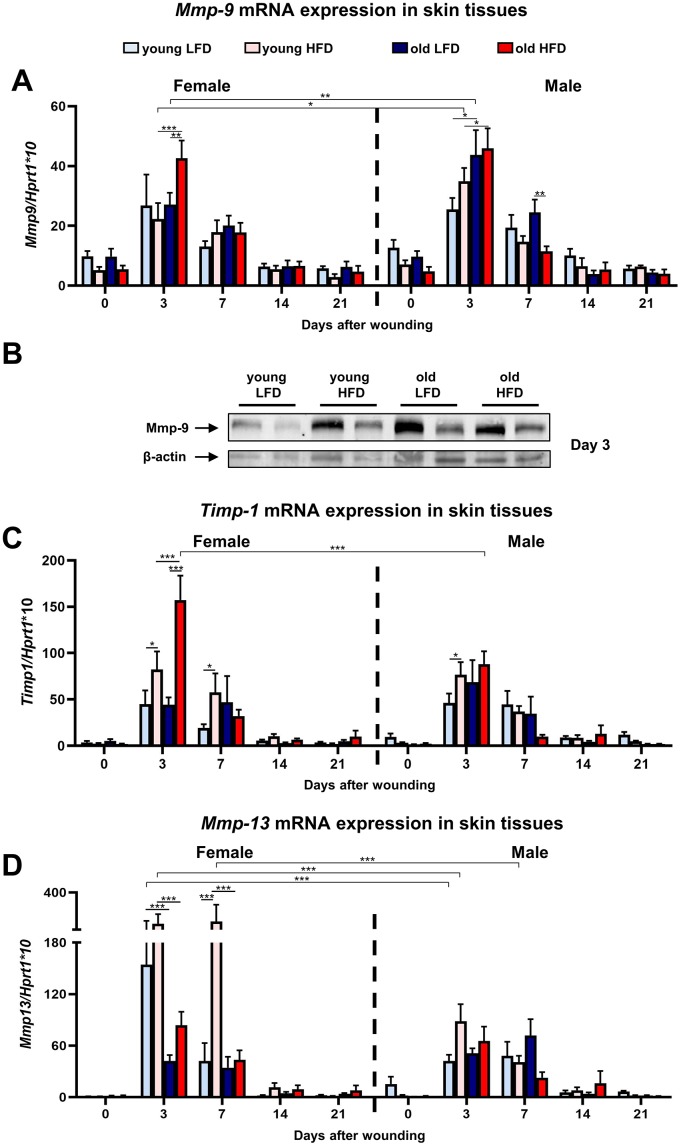
**Matrix metalloproteinases and their tissue inhibitor expression during skin wound healing.** *Mmp-9* (**A**), *Timp-1* (**C**) and *Mmp-13* (**D**) qRT-PCR mRNA expression in uninjured and post-injured skin tissues collected from female, male, young, old, fed LFD or HFD mice (n=4-8 skin samples per group). Representative Western blot analysis of Mmp-9 protein expression at post-wounded day 3 (**B**). The bars indicate lsmean ±SE *p<0.05, **p<0.01, ***p<0.001.

The skin wound healing process is tightly coordinated by Mmps enzymes [31]. Mmps function as regulators of each phase of skin wound healing contributing to the inflammatory and remodelling stages through their participation in the degradation of structural extracellular matrix (ECM) components, activation of other Mmps, release of growth factors from the cell membrane or ECM, and the shedding of cell adhesion molecules [32]. The analysis of *Mmp-9* (Figure 5A; Supplementary Tables 23–26) and *Timp-1* (Figure 5C; Supplementary Tables 27–30) revealed a surge in mRNA expression in post-injured skin tissues at day 3 for both males and females followed by their gradual decrease to reach the initial baseline (day 0) levels at day 14. The highest levels of *Mmp-9* mRNA expression at post wounded day 3 was observed for old HFD mice, indicating the impact of two parameters: age and diet (Figure 5A; Supplementary Tables 23–26). The surge in *Mmp-9* expression at day 3 accompanied a burst of *Timp-1* mRNA where *Timp-1* expression was higher for old HFD females relative to males (Figure 5C; Supplementary Tables 27–30). The combined effect of: diet, age and sex was detected at day 3^rd^ after wounding for *Timp-1* mRNA surge (Figure 5C; Supplementary Tables 27–30). The analysis of Mmp-9 protein expression at day 3 by Western blot confirmed that age and HFD diet stimulated its expression (Figure 5B).

Likewise, Mmp-13 is a metalloproteinase which regulates skin wound healing through participation in the growth of granulation tissue, organization of myofibroblasts, and the formation of large blood vessels [33]. Similar to *Mmp-9*, peak *Mmp-13* mRNA expression for both females and males was observed in samples collected at day 3 and day 7 after injury (Figure 5D). In contrast to *Mmp-9* (compare Figure 5A), the levels of *Mmp-13* expression at post-injured day 3 and 7 was the highest in young HFD females (Figure 5D; Supplementary Tables 31–34). The two variables: age and sex at day 3^rd^ and three variables: age, sex and diet at day 7 act in combination to increase the expression of *Mmp-13* mRNA (Figure 5D; Supplementary Tables 31–34). At post-wounded days 14 and day 21 the expression of *Mmp-13* decreased and approached the baseline levels observed in non-injured skin samples (Figure 5D).

**Figure 6:**
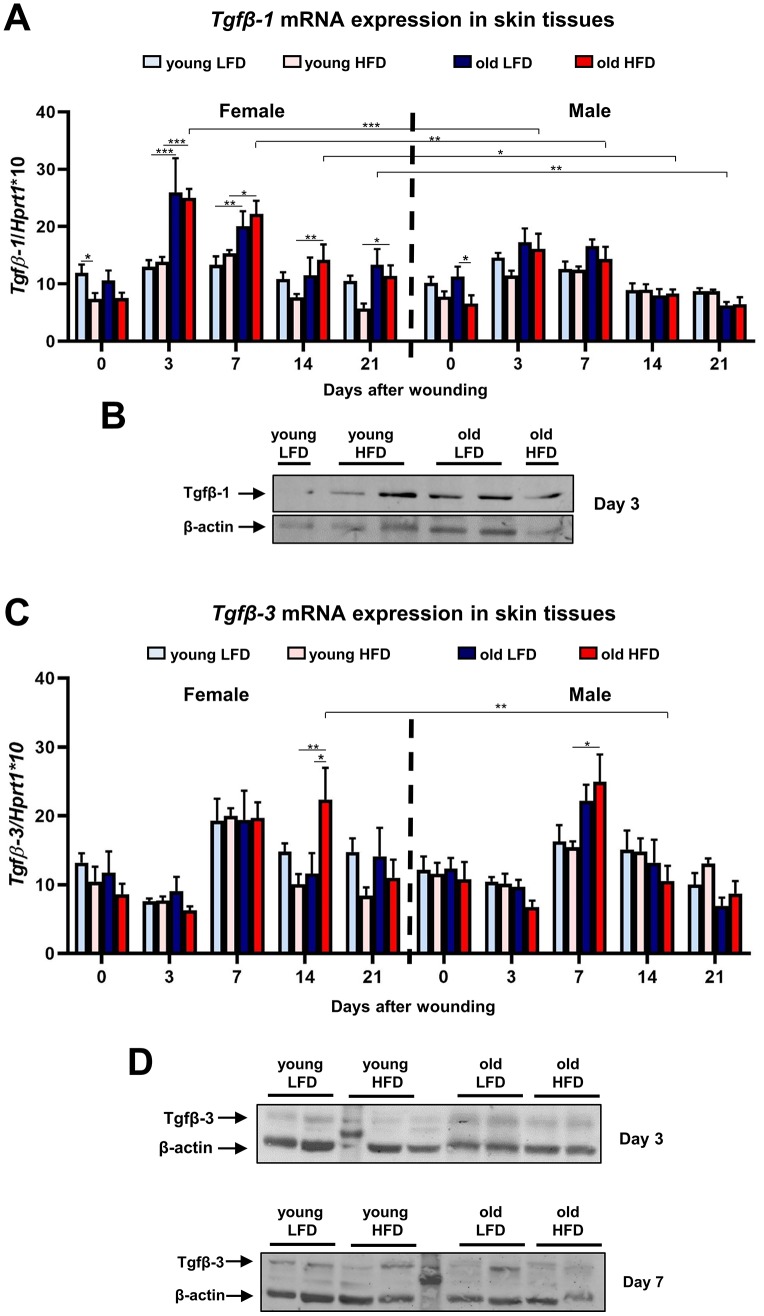
**Tgfβ-1 and Tgfβ-3 expression during skin wound healing.** qRT-PCR mRNA expression of *Tgfβ-1* (**A**) and *Tgfβ-3* (**C**) in uninjured and post-injured skin tissues collected from female, male, young, old, fed LFD or HFD mice (n=4-8 skin samples per group). Representative Western blot analysis of Tgfβ-1 (**B**) and Tgfβ-3 (**D**) protein expression at post-wounded day 3 (**B**, **D**) and day 7 (**D**). The bars indicate lsmean ±SE *p<0.05, **p<0.01, ***p<0.001.

Other factors that affect the skin wound healing process are transforming growth factors beta 1 and 3 (Tgfβ-1 and Tgfβ-3). Tgfβ-1, so called pro-scarring, is one of the first cytokines to elicit inflammatory cell recruitment. The skin wound healing process evokes Tgfβ-1 mRNA (Figure 6A) and protein (Figure 6B) expression at post-wounded days 3 and 7. The highest expression of *Tgfβ-1* was detected in post-wounded skin from old female mice regardless of diet (Figure 6A). The upregulated *Tgfβ-1* gene expression at day 3 (p<0.001), 7 (p<0.01) and 14 (p<0.05) is substantially greater in old HFD females than relative to old HFD males (Figure 6A; Supplementary Tables 35–38). The expression levels of Tgfβ-3, a cytokine which plays an important role in wound repair and is presumed to be a potential mediator of scar reduction and skin healing improvement [34], showed the increase for both females and males at post-wounding day 7 (Figure 6C, 6D; Supplementary Tables 39–42). For old HFD females *Tgfβ-3* mRNA expression remained elevated at day 14 after injury (Figure 6C) indicating the interaction among: sex (female) and diet (HFD) and post-wounding day (day 14) (Supplementary Table 42).

**Figure 7:**
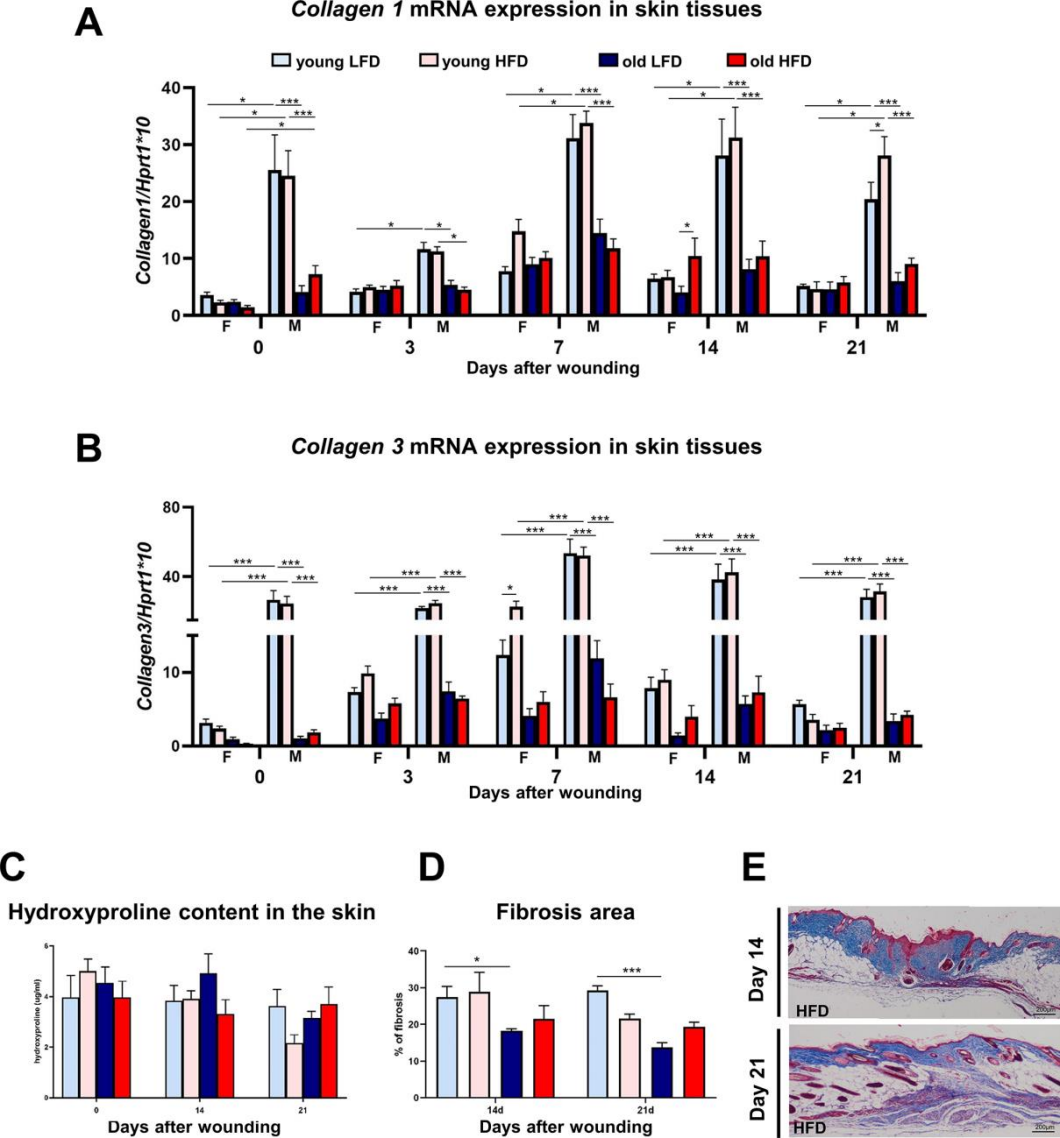
**Collagen expression, hydroxyproline content and fibrosis localization in uninjured and post-injured skin.** *Collagen 1* (**A**) and *collagen 3* (**B**) mRNA expression (n=4-8 skin samples per group) in skin tissues of old, young, male (M), female (F), LFD or HFD mice. Hydroxyproline (**C**) content (n=4-8 skin samples per group) in skin tissues of old, young, LFD or HFD mice. Histological skin sections stained with Masson trichrome collected from young or old mice fed LFD or HFD at post-injured day 14 and 21 (**D**), followed by quantification of the fibrosis area (**E**) (n=3 mice per group). Scale bar (**E**) 200 μm. The bars indicate lsmean ±SE *p<0.05, **p<0.01, ***p<0.001.

The restoration of post-injured skin tissues requires collagen biosynthesis and turnover that affect the post-wounded skin quality and outcome. To evaluate collagen content during skin wound healing, we analyzed *collagen 1* and *3* mRNA expression, total collagen content through hydroxyproline assays, and analysis of post-wounded area on Masson’s trichrome stained histological sections (Figure 7). The highest levels of *collagen 1* and *3* mRNA in uninjured and post-injured skin tissue samples were detected in young male mice (Figure 7A, 7B, Supplementary Tables 43–50) whereas the expression in aged males was significantly lower and comparable to young and old females (Figure 7A, 7B; Supplementary Tables 43–50). Evidently, age (young) and sex (males) but not diet have the combined effect on the *collagen1* and *3* gene expression in the skin (Figure 7A–7B; Supplementary Table 46–50). Further analysis of collagen was performed through hydroxyproline content measurement (Figure 7C) and the histological analysis of Masson’s Trichrome stained skin sections (Figure 7D, 7E) at post-wounded day 14 and 21. The combined effect of age and diet for hydroxyproline content was detected at day 21 after wounding (Supplementary Table 51). The highest percentage of fibrosis area analyzed in histological sections was quantified for young and low fat diet mice at both day 14 and 21 (Figure 7D, 7E; Supplementary Tables 52, 53).

## DISCUSSION

In this study, we have compared the skin wound healing process as a function of age (younger vs older), sex (females vs males) and diet (LFD vs HFD) in C57BL/6 mice in order to determine the cumulative effect of age, sex and diet on cutaneous wound healing.

Overall, our data indicate that although all three components: age, sex and diet affect the quality of the skin before and after wounding, it is age that has the most fundamental impact (Table 1; Supplementary Tables 1–53). First of all, older mice displayed a greater body fat mass accumulation when placed in an obesogenic environment accompanied by an increased reduction in the thickness of the fibroblast-rich dermis in the intact skin. Skin wound healing at particular stages was affected by age in the aspect of Tgfβ-1 (days 3, 7 and 21); MCP-1 (day 3), Mmp-9 (day 3) and Mmp-13 (days 3, 7) expression and hydroxyproline content (day 21) (Table 1).

**Table 1:** The summary of the impact/interactions of age, diet, sex on skin wound healing in young or old, fed the LFD or HFD, females or males B6 mice based on coefficients of model for factors affecting the particular days of the process. Impact/interactions is arranged from the most to the least (1, 2, 3) influential for each factor.

**Day of wound healing**	**Factor/process**	**Impact/Interactions**
**Day 3**	MCP-1	Age
	*Mmp-9* mRNA expression	1. Age
		2. Diet
	*Timp-1* mRNA expression	1. Diet
		2. Sex:diet
		3. Age:sex:diet
	*Tgfβ-1* mRNA expression	1. Age
		2. Sex
	*Mmp-13* mRNA expression	1. Age
		2. Age:sex
**Day 7**	*Tgfβ-1* mRNA expression	1. Age
		2. Sex
		3. Diet
	*Mmp-13* mRNA expression	1. Age
		2. Sex:diet
		3. Age:diet
		4. Age:sex
**Day 14**	*Tgfβ-1* mRNA expression	Diet
	*Tgfβ-3* mRNA expression	1. Age:sex
		2. Sex
**Day 21**	*Tgfβ-1 mRNA expression*	1. Age
		2. Age:sex
	Hydroxyproline content	1. Age
		2. Age:diet
**Days 3, 7, 14 and 21**	*CD68* mRNA expression	Age:diet
	*Collagen 1* mRNA expression	Age:sex
	*Collagen 3* mRNA expression	Age:sex
**Days 3 and 7**	Re-epithelialization	Age:diet

The most pronounced cumulative effect was observed for the combination of two parameters: age and sex. The effect predominantly was detected for *collagen 1* and *collagen 3* expression which was extremely high in younger males before injury and throughout the entire process of skin wound healing (Tables 1). Likewise, the combined effect of age and sex impacted *Mmp-13* expression at day 3 and day 7 after injury was significantly higher for younger females. Diet as a single variable modified the thickness of dermis as a result of increased dWAT accumulation in animals fed HFD. In injured skin, diet had an impact on the expression of *Timp-1* (day 3), and *Tgfβ-1* (day 14). The combination of age and diet affected the re-epithelialization process and inflammatory response based on *CD68* mRNA expression during the skin wound healing process. Interestingly, the only cumulative effect combining three parameters (age, diet and sex) on the cutaneous wound healing process was displayed exclusively by *Timp-1* mRNA for which high levels were observed for older HFD females at day 3 after wounding (Table 1).

The fastest rate of wound re-epithelialization was detected in young LFD mice (63.4% ±11.5) at day 3^rd^ that was substantially reduced by age (25.9%±14.0) in old LFD mice. Komi-Kuramochi et al. [35], comparing the 6 mm wound closure between young (8-weeks) vs old (35-weeks) mice, found the rate of 50% at post-wounded day 3^rd^ for young mice. Similarly, a comparably fast rate of 3 mm skin wound re-epithelialization by day 3^rd^ for young mice was also detected for C57BL/6 (79±14%) [26] and BALB/c (60%) [36] mice regardless genetic background.

The growth factor TGFβ comprises three isoforms: TGFβ-1, TGFβ-2, TGFβ-3 which modulate skin and cutaneous wound healing. Two isoforms, TGFβ-1 and TGFβ-2, attract neutrophils, macrophages and fibroblasts into the wound [37], contribute to the resolution of inflammation [38] and orchestrate re-epithelialization, angiogenesis and granulation tissue formation [39–41]. TGFβ-1 and TGFβ-2 promote fibroblasts differentiation into their active state as myofibroblasts, which regulate connective tissue remodelling by combining production of ECM components with characteristics of contractile smooth muscle cells [42–44]. During the last phase of cutaneous wound repair, remodelling, TGFβ-1 and 2, together with Mmps and Timps regulate collagen production and degradation, leading to formation of mature scar [1]. While TGFβ-1 and TGFβ-2 promote scar formation, TGFβ-3 acts as anti-fibrotic agent. This cytokine appears early after injury, particularly in the migrating epidermis [45] and is present in the neo-epidermis when re-epithelialization is completed. Experiments performed by Le et al [46] have shown that although TGFβ-3 does not promote re-epithelialization, it is necessary for wound closure. Data obtained by Shah et. al [47] indicates that exogenous addition of neutralizing antibody to TGFβ-1 and TGFβ-2 reduces scarring. Similar results were obtained after exogenous addition of the TGFβ-3 peptide to the wound what caused diminished scarring and improved the architecture of the scar. In our studies, we analyzed the expression profile of Tgfβ-1 and Tgfβ-3 during cutaneous wound healing. The peak of Tgfβ-1 was observed at day 3 and 7 after injury with higher expression in post-wounded skin tissues collected from old mice. Increased levels of Tgfβ-1 in aged mice 3 and 7 days after wounding corresponded to an enhanced inflammatory response characterized by a higher level of MCP-1 and increased macrophage infiltration. The expression pattern of Tgfβ-3, a potential mediator of scar reduction and skin healing improvement [34], varied significantly depending on age and sex, that was particularly detected at post-wounding day 14. According to our knowledge, this is the first report describing the differential expression profile of Tgfβ-3 during skin wound healing process between young and aged mice. Further studies exploring the impact of biological aging on this skin wound biomarker are warranted.

Since Tgfβ isoforms are involved in the ECM synthesis by upregulating multiple extracellular matrix genes including collagens, [48], altered levels of circulating Tgfβ in old animals can modify the collagen content in the post-injured skin of aged individuals. This is consistent with our results which showed much higher levels of *collagen 1* and *3* mRNA in the skin of young male mice when compared to old mice of matched sex. The analysis of transcriptome data published by Salzer et al. performed on fibroblasts isolated from aged mice further confirms the decreased expression of ECM genes including collagens and glycosaminoglycans [49]. Our studies of collagen content showed differences in total skin concentration between male and female mice, independent of age-related changes. The data collected from multiple experiments suggest that sex steroids may account for the difference in the skin collagen content between males and females. While a series of studies have identified estrogens as enhancers of healing, androgens (testosterone and its metabolite 5α-dihydrotestosterone (DHT) are postulated to serve as negative regulators of the repair process [50, 51].

The main aim of our study was to define the combined impact of age, sex and diet on skin characteristics. We observed that young females fed HFD showed the levels of *Mmp-13* mRNA expression at post-wounded day 7 that were more than four times higher than in any other groups of animals. While the collagenase Mmp-13 is almost undetectable in intact mouse skin, its expression is induced by injury [52]. Two cell types express Mmp-13 during the early phase of cutaneous healing: keratinocytes at the leading epithelial tongue of neoepidermis [53] and dermal fibroblasts, regulating angiogenesis, proteolysis and myofibroblasts motility during granulation tissue formation [33]. Several studies indicate that adipocytes may serve as another source of Mmp-13. Unpublished data by Shih et al. has suggested that epididymal white adipose tissue of mice fed HFD express Mmp-13 [54]. Data by Ezure and Amano demonstrated that co-culture of enlarged adipocytes with 3T3-L1 fibroblasts significantly increased the mRNA levels of *Mmp-13* [7]. Results published by Schmidt and Horsley indicated that during the proliferative phase of healing, at day 5 after injury, small adipocytes repopulate skin wounds [55]. In our study, high mRNA levels of *Mmp-13* detected in young females fed HFD may originate not only from keratinocytes and fibroblasts but also from repopulating intradermal adipocytes. dWAT, the separate layer presented within reticular dermis, was defined initially by Wojciechowicz et al. [23]. Since then multiple reports have linked the dWAT to the inflammatory response [25], thermogenesis [56, 57], hair cycling [58–61], aging [62, 63], wound healing, fibrosis and scarring [55, 64].

The contribution of dWAT in the skin wound healing process in old HFD mice warrants additional attention. However, accumulating evidence suggests that the presence of dermal adipocytes during cutaneous injury repair may be beneficial for the healing outcome. In current studies, we showed that mice fed HFD displayed thicker dermis than animals fed LFD, regardless of age. The increase in dWAT thickness, as a result of HFD, may have compensated for the age-related loss in fibroblasts-rich dermis. Moreover, in old HFD mice we observed the tendency towards a faster rate of re-epithelialization comparing to old LFD animals. Experiments performed by Schmidt and Horsley [55] confirmed the role of dermal adipocytes in injury repair by showing that the lack of adipocytes in the wound leads to invalid dermal remodelling, skin failure, and reopening of the wound. A recently described feature of skin adipocytes, which may prove crucial for cutaneous healing, is their plasticity, after the exposure to pro-fibrotic agents such as TGFβ, characterized by their ability to transition into myofibroblasts (adipocyte-myofibroblasts transition -AMT) [64], and reprogramming of myofibroblasts into adipocytes by activation the BMP2 pathway [60].

In conclusion, our data in a murine model have shown that while diet and sex have considerable impact on skin wound healing, advancing age exerts the most profound effect. These findings suggest that further mechanistic studies exploring the dynamics between these biological variables in pre-clinical animal models and in human subjects are warranted.

## MATERIALS AND METHODS

### Animals

The experimental animal procedures performed in these studies were approved by the Ethics Committee of University of Warmia and Mazury (Olsztyn, Poland), No. 22/2015.

The C57BL/6J (B6) mice for the study were generated through colonies established at the animal facility in Institute of Animal Reproduction and Food Research of Polish Academy of Sciences. B6 mice were originally purchased from The Jackson Laboratory (Bar Harbor, ME USA). At the beginning of the experiment, adult (2-3 months), and old (16-18 months), male and female (young; n=24 per group and old; n=16-24 per group) B6 mice were assigned into separated groups fed for a period of 8 weeks either a low fat diet (LFD, 13 kcal% fat; PicoLab Rodent Diet 20 5053) or high fat diet (HFD 59 kcal% fat; TestDiet AIN-76A; LabDiet) (scheme of the experiment Fig. 1). At the end of every week of a diet (LFD or HFD), body mass was measured and body composition was analyzed. Measurements of lean and fat mass were acquired by nuclear magnetic resonance (NMR) using the Minispec LF90II (Bruker Optics). After 8 weeks of diet, glucose tolerance test (GTT) was performed, as described by Anunciado-Koza et al. [65]. Briefly, after fasting for 4 h from 8:00 to 12:00, mice received an intraperitoneal injection of 20% glucose solution (2 g/kg of body weight; Sigma-Aldrich by Merck). Blood glucose levels were determined using a blood glucose monitor (Accu-chek, Roche).

### Wounding and collecting skin tissue samples

The day before wounding 5 months old (young) and 21 months old (old) female and male B6 mice, fed LFD or HFD were anesthetized with isoflurane and shaved in the dorsal area. On the following day (day 0), mice were anesthetized with isoflurane and four full-thickness excisional wounds were created on the back of mice using a 4 mm biopsy punch (Miltex). Excised skin samples (4 mm in diameter) were collected, immediately frozen in liquid nitrogen and stored at -80°C until analysis (day 0, uninjured control). B6 mice were sacrificed at days 3, 7, 14 and 21 after wounding. Post-injured skin areas were collected with 8 mm diameter biopsy punches. Tissue samples (n=6-8 per time point) for RNA or protein isolation were immediately frozen in liquid nitrogen and stored in -80°C till further use. Samples for histology were fixed in formalin (n=3-6 per time point).

### Masson trichrome staining, histological measurement of the thickness of layers of the skin and analysis of fibrosis

Formalin-fixed sections of uninjured and post-injured skin tissues were deparaffinized by immersion in xylene and then rehydrated. The samples were stained with Trichrome Stain Kit (Modified Masson's) (ScyTek Laboratories, Inc.) For the histological analysis of uninjured skin and measurements of fibrosis in post-injured skin tissues, Image J image analysis software was used (National Institutes of Health (NIH) Image).

### Analysis of re-epithelialization

The percentage of re-epithelialization was calculated as previously described by Low et al. [26] and Noguchi et al. [66]. Briefly, the width of wounds and the distance of migrating epithelium were measured on post-injured skin sections (days 3, 7, 14 after injury) stained with hematoxylin and eosin (H&E). The percentage of re-epithelialization was calculated according to the formula: [length of the extending epidermal tongues]/[length of the wound] x100% (number of mice n = 3-5 per group, number of measurements n = 3-10 per group) Measurements were performed using ImageJ software (NIH Image).

### Monocyte chemotactic protein 1 (MCP-1) detection assay

The ELISA assay for quantitative determination of MCP-1 concentrations in tissue homogenates was performed according to the manufacturer’s protocol (Cusabio). Collected skin tissue samples from day 0 (controls), and post-wounded day 3 and day 7 were weighed before further preparation. The samples were homogenized in PBS using 100 mg tissue per 1ml of PBS. The average weight of skin tissue samples varied between 30 and 80 mg therefore an appropriately adjusted volume (between 0.3 to 0.8 ml) of PBS was used for each tissue sample. Tissue homogenates were stored overnight at -20°C. After two freeze and thaw cycles, the homogenates were centrifuged (5 minutes/5000 x g, 2-8°C) and the supernatant was collected, aliquoted and stored at -80°C until performing the assay.

### Immunohistochemistry

Formalin-fixed sections of post-injured (day 3 and 7) skin tissues were processed, embedded in paraffin and sectioned. Slides were stained with Masson’s trichrome (Trichrome Stain Kit, Abcam) for collagen presence using standard protocol. Immunohistochemical staining for the presence of Mac-2 was performed with anti- LGALS3 (1:2500, mouse monoclonal, LSBio), CD68 (1:200, rabbit polyclonal, Abcam) and Cytokeratin 16 (1:300, rabbit polyclonal, LsBio). Antibody binding was detected with the ABC complex (Vectastain ABC kit, Vector Laboratories, Inc.). In control sections primary antibodies were substituted with non-specific-immunoglobulin G (IgG). Peroxidase activity was revealed using 3.3′-diaminobenzidine (Sigma-Aldrich by Merck) as a substrate. Slides were counterstained with hematoxylin (Sigma-Aldrich by Merck). Sections were visualized using Olympus microscope (BX43), photographed with Olympus digital camera (XC50) and analyzed with Olympus CellSens Software.

### RNA isolation and quantitative RT-PCR

Total RNA was extracted from skin samples using TRI Reagent (Sigma-Aldrich by Merck) according to the manufacturer’s instructions. Quantity and quality of RNA was verified on NanoDrop 1000 (Thermo Fisher Scientific) and through analysis of agarose gel after electrophoresis. cDNA was synthesized from 500 ng of total RNA using High-Capacity cDNA Reverse Transcription Kit with RNase Inhibitor (Applied Biosystems by Thermo Fisher Scientific). To measure the levels of *Collagen 1, Collagen 3, Hprt-1, Mmp-9, Mmp-13, Tgfβ-1, Tgfβ-3* and *Timp-1,* mRNA expression, Single Tube TaqMan® Gene Expression Assays (Life Technologies Thermo Fisher Scientific) were used. Amplification was performed using 7900HT Fast Real-Time PCR System under conditions: initial denaturation for 10 min at 95°C, followed by 40 cycles of 15 sec at 95°C and 1 min at 60°C. Each run included standard curve based on aliquots of pooled skin RNA. All samples were analyzed in duplicates. *Hprt-1* was chosen as the most stable housekeeping gene during cutaneous wound healing after analysis described previously [67]. mRNA expression levels were normalized to the reference gene *Hprt-1* and multiplied by 10.

### Protein isolation and western blot analysis

Frozen post-injured skin samples collected at day 0, 3 and 7 were homogenized in liquid nitrogen with mortar and pestle in 500 μl of RIPA buffer containing protease inhibitor cocktail (Sigma-Aldrich by Merck), Phosphatase inhibitor cocktail (Sigma-Aldrich by Merck) and Phenylmethanesulfonyl fluoride (PMSF, Sigma-Aldrich by Merck) further sonicated (3 × 20 s, 20 kHz) with Vibro-Cell VCX 130 PB ultrasound sonicator (Sonics). Protein concentration was measured by the infrared (IR)-based protein quantitation method using a Direct Detect® Infrared Spectrometer (Merck) as previously described [68]. Thirty micrograms of proteins were separated on 12% SDS-polyacrylamide gels and transferred onto polyvinylidene difluoride membranes (Merck Millipore). The membranes were incubated separately with antibodies: anti- Mmp-9 (1:1000, rabbit polyclonal, Merck Millipore), anti-Tgfβ-1 (1:500, rabbit polyclonal, Biorbyt), anti-Tgfβ-3 (1:200, rabbit polyclonal, Abcam) and anti-β-actin (1:1000 mouse monoclonal, Abcam), followed by fluorescent secondary antibodies IRDye 800 (1:5000 goat anti-rabbit, Rockland) and Cy5.5 (1:10000, goat anti-mouse, Rockland). Bands were visualized using the ChemiDoc™ Touch Imaging System (Bio-Rad) and analyzed using Image Lab Software (Bio-Rad) according to the manufacturer’s protocol.

### Measurement of hydroxyproline

Collagen assay was prepared as described before [69]. Briefly, 8 mm diameter frozen skin punches, were homogenized in 2 ml of phosphate buffered saline (Sigma-Aldrich by Merck) and stored in 4 °C overnight. The next day, 0.5 ml aliquots were hydrolyzed with 0.25 ml of 6N HCL for 4.5 h at 120 °C. To construct a standard curve hydroxyproline concentrations from 0 to 20 μg/ml were used (Sigma-Aldrich by Merck). 20 μl of each sample and standard curve point was added to a 96 well plate and incubated for 20 min at room temperature with 50 μl of chloramine T solution (282 mg chloramine T, 2 ml n-propanol, 2 ml distilled water and 16 ml citrate acetate buffer [5% citric acid, 7.24% sodium acetate, 3.4% sodium hydroxide and 1.2% glacial acetic acid]). Next, 50 μl of Ehrlich's solution (2.5 g 4-(dimethyloamino) benzaldehyde, 9.3 ml n-propanol and 3.9 ml 70% perchloric acid) was added and the plate was incubated for 15 min at 65°C. After cooling down the samples the plate was read at 550 nm on a microplate reader (Multiskan Sky Microplate Spectrophotometer, Thermo Fisher Scientific™).

### Statistical analysis

In order to analyze impact of gender, age, diet and timepoint on body parameters linear mixed-effects models were used, in which mice were included as random intercept and mentioned factors and all possible interactions were included as fixed effects. Similarly, mixed-effect models were prepared for skin tissues parameters; however the random effects were insignificant, so linear models were used instead. Based on such models, lsmeans were calculated (least-squared means - means calculated based on model’s coefficients) and compared (for mixed-effects models Kenward-Roger method for degrees of freedom was used; Tukey p-value adjustment used where appropriate). Results are presented in tables and plots (lsmeans with standard error). Moreover, optimal models were obtained using backward stepwise method. All calculations were performed in R (ver. 3.5.3) using packages: lme4, lmerTest, emmeans and tidyverse.

## Supplementary Material

Supplementary Tables
