# Supplementary material for: Cutaneous wound healing in aged, high fat diet-induced obese female or male C57BL/6 mice
Source: Aging (Albany NY). 2020 Apr 15;12(8):7066–111. doi: 10.18632/aging.103064 (PMC7202484; doi:10.18632/aging.103064)
Supplement: Supplementary Tables [file aging-12-103064-s001..pdf]

SUPPLEMENTARY TABLES

Statistically significant differences for body weight by diet, sex and age

Supplementary Table 1. Comparisons by diet for body weight.

diet	age	sex	timepoint	lsmean	lower.CL	upper.CL	SE	df	p.value
HFD	old	female	W3	33.6	31.4	35.8	1.1	873.4	0.013
LFD	old	female	W3	29.4	26.8	32.0	1.3	1005.6	
HFD	old	female	W4	35.6	34.0	37.3	0.9	581.6	<0.001
LFD	old	female	W4	30.5	28.3	32.6	1.1	873.3	
HFD	old	female	W5	35.3	33.6	37.0	0.9	616.8	0.001
LFD	old	female	W5	31.0	28.8	33.1	1.1	873.3	
HFD	old	female	W6	36.6	34.9	38.4	0.9	616.8	<0.001
LFD	old	female	W6	31.3	29.2	33.5	1.1	873.3	
HFD	old	female	W7	36.9	35.2	38.6	0.9	616.8	<0.001
LFD	old	female	W7	31.2	28.8	33.6	1.2	964.2	
HFD	old	female	W8	37.7	36.0	39.4	0.9	581.6	<0.001
LFD	old	female	W8	30.7	28.8	32.6	1.0	736.8	
HFD	old	male	W1	37.4	35.4	39.3	1.0	781.0	0.011
LFD	old	male	W1	33.9	31.8	35.9	1.0	826.8	
HFD	old	male	W2	39.0	37.1	41.0	1.0	781.0	<0.001
LFD	old	male	W2	33.7	31.7	35.8	1.0	826.8	
HFD	old	male	W3	40.7	38.8	42.7	1.0	781.0	<0.001
LFD	old	male	W3	34.0	31.9	36.1	1.0	826.8	
HFD	old	male	W4	44.3	42.8	45.8	0.8	440.0	<0.001
LFD	old	male	W4	34.3	32.6	36.0	0.9	581.6	
HFD	old	male	W5	45.2	43.7	46.7	0.8	440.0	<0.001
LFD	old	male	W5	34.5	32.6	36.4	1.0	736.8	
HFD	old	male	W6	46.2	44.7	47.7	0.8	440.0	<0.001
LFD	old	male	W6	34.6	32.7	36.4	1.0	736.8	
HFD	old	male	W7	46.8	45.3	48.3	0.8	464.3	<0.001
LFD	old	male	W7	34.3	32.6	35.9	0.9	581.6	
HFD	old	male	W8	47.4	45.8	48.9	0.8	490.5	<0.001
LFD	old	male	W8	34.6	32.9	36.3	0.9	581.6	
HFD	young	female	W4	23.2	21.7	24.7	0.8	440.0	0.039
LFD	young	female	W4	21.2	19.8	22.7	0.7	400.4	
HFD	young	female	W5	23.5	22.0	25.0	0.8	440.0	0.036
LFD	young	female	W5	21.5	20.1	22.9	0.7	400.4	
HFD	young	female	W6	25.3	23.8	26.8	0.8	440.0	<0.001
LFD	young	female	W6	21.8	20.3	23.2	0.7	400.4	
HFD	young	female	W7	25.2	23.7	26.7	0.8	440.0	<0.001
LFD	young	female	W7	21.7	20.3	23.1	0.7	400.4	
HFD	young	female	W8	26.7	24.9	28.6	0.9	695.1	<0.001
LFD	young	female	W8	22.7	21.0	24.4	0.9	616.8	
HFD	young	male	W2	27.2	25.6	28.9	0.8	548.8	0.029
LFD	young	male	W2	25.0	23.4	26.5	0.8	464.1	
HFD	young	male	W3	28.4	26.7	30.0	0.8	548.8	0.009
LFD	young	male	W3	25.7	24.2	27.2	0.8	464.1	

HFD	young	male	W4	30.2	28.6	31.9	0.8	548.8	<0.001
LFD	young	male	W4	26.0	24.5	27.5	0.8	464.1	
HFD	young	male	W5	30.9	29.2	32.5	0.8	548.8	<0.001
LFD	young	male	W5	26.5	25.0	28.0	0.8	464.1	
HFD	young	male	W6	33.4	31.8	35.1	0.8	548.8	<0.001
LFD	young	male	W6	26.8	25.2	28.3	0.8	464.1	
HFD	young	male	W7	34.0	32.4	35.6	0.8	548.8	<0.001
LFD	young	male	W7	27.0	25.5	28.6	0.8	464.1	
HFD	young	male	W8	34.5	32.4	36.6	1.0	826.9	<0.001
LFD	young	male	W8	27.2	25.2	29.1	1.0	781.3	

Supplementary Table 2. Comparisons by sex for body weight.

sex	diet	age	timepoint	lsmean	lower.CL	upper.CL	SE	df	p.value
female	HFD	old	W0	30.3	28.6	32.0	0.9	581.6	<0.001
male	HFD	old	W0	36.8	35.3	38.3	0.8	440.0	
female	HFD	old	W1	30.8	28.7	33.0	1.1	873.4	<0.001
male	HFD	old	W1	37.4	35.4	39.3	1.0	781.0	
female	HFD	old	W2	31.4	29.2	33.7	1.2	919.6	<0.001
male	HFD	old	W2	39.0	37.1	41.0	1.0	781.0	
female	HFD	old	W3	33.6	31.4	35.8	1.1	873.4	<0.001
male	HFD	old	W3	40.7	38.8	42.7	1.0	781.0	
female	HFD	old	W4	35.6	34.0	37.3	0.9	581.6	<0.001
male	HFD	old	W4	44.3	42.8	45.8	0.8	440.0	
female	HFD	old	W5	35.3	33.6	37.0	0.9	616.8	<0.001
male	HFD	old	W5	45.2	43.7	46.7	0.8	440.0	
female	HFD	old	W6	36.6	34.9	38.4	0.9	616.8	<0.001
male	HFD	old	W6	46.2	44.7	47.7	0.8	440.0	
female	HFD	old	W7	36.9	35.2	38.6	0.9	616.8	<0.001
male	HFD	old	W7	46.8	45.3	48.3	0.8	464.3	
female	HFD	old	W8	37.7	36.0	39.4	0.9	581.6	<0.001
male	HFD	old	W8	47.4	45.8	48.9	0.8	490.5	
female	LFD	old	W0	30.0	28.1	31.9	1.0	736.8	<0.001
male	LFD	old	W0	35.2	33.5	36.8	0.9	581.6	
female	LFD	old	W1	29.2	27.0	31.4	1.1	873.3	0.001
male	LFD	old	W1	33.9	31.8	35.9	1.0	826.8	
female	LFD	old	W2	29.8	27.7	32.0	1.1	873.3	0.007
male	LFD	old	W2	33.7	31.7	35.8	1.0	826.8	
female	LFD	old	W3	29.4	26.8	32.0	1.3	1005.6	0.005
male	LFD	old	W3	34.0	31.9	36.1	1.0	826.8	
female	LFD	old	W4	30.5	28.3	32.6	1.1	873.3	0.003
male	LFD	old	W4	34.3	32.6	36.0	0.9	581.6	
female	LFD	old	W5	31.0	28.8	33.1	1.1	873.3	0.010
male	LFD	old	W5	34.5	32.6	36.4	1.0	736.8	
female	LFD	old	W6	31.3	29.2	33.5	1.1	873.3	0.020
male	LFD	old	W6	34.6	32.7	36.4	1.0	736.8	
female	LFD	old	W7	31.2	28.8	33.6	1.2	964.2	0.033
male	LFD	old	W7	34.3	32.6	35.9	0.9	581.6	

female	LFD	old	W8	30.7	28.8	32.6	1.0	736.8	0.001
male	LFD	old	W8	34.6	32.9	36.3	0.9	581.6	
female	HFD	young	W0	19.5	18.0	21.0	0.8	440.0	<0.001
male	HFD	young	W0	24.0	22.4	25.6	0.8	548.8	
female	HFD	young	W1	19.9	18.5	21.4	0.8	440.0	<0.001
male	HFD	young	W1	25.9	24.2	27.5	0.8	548.8	
female	HFD	young	W2	21.7	20.2	23.2	0.8	440.0	<0.001
male	HFD	young	W2	27.2	25.6	28.9	0.8	548.8	
female	HFD	young	W3	22.4	20.9	23.9	0.8	440.0	<0.001
male	HFD	young	W3	28.4	26.7	30.0	0.8	548.8	
female	HFD	young	W4	23.2	21.7	24.7	0.8	440.0	<0.001
male	HFD	young	W4	30.2	28.6	31.9	0.8	548.8	
female	HFD	young	W5	23.5	22.0	25.0	0.8	440.0	<0.001
male	HFD	young	W5	30.9	29.2	32.5	0.8	548.8	
female	HFD	young	W6	25.3	23.8	26.8	0.8	440.0	<0.001
male	HFD	young	W6	33.4	31.8	35.1	0.8	548.8	
female	HFD	young	W7	25.2	23.7	26.7	0.8	440.0	<0.001
male	HFD	young	W7	34.0	32.4	35.6	0.8	548.8	
female	HFD	young	W8	26.7	24.9	28.6	0.9	695.1	<0.001
male	HFD	young	W8	34.5	32.4	36.6	1.0	826.9	
female	LFD	young	W0	19.7	18.2	21.1	0.7	400.4	<0.001
male	LFD	young	W0	23.4	21.8	24.9	0.8	464.1	
female	LFD	young	W1	20.3	18.9	21.7	0.7	400.4	<0.001
male	LFD	young	W1	24.4	22.9	25.9	0.8	464.1	
female	LFD	young	W2	20.6	19.2	22.0	0.7	400.4	<0.001
male	LFD	young	W2	25.0	23.4	26.5	0.8	464.1	
female	LFD	young	W3	21.0	19.6	22.5	0.7	400.4	<0.001
male	LFD	young	W3	25.7	24.2	27.2	0.8	464.1	
female	LFD	young	W4	21.2	19.8	22.7	0.7	400.4	<0.001
male	LFD	young	W4	26.0	24.5	27.5	0.8	464.1	
female	LFD	young	W5	21.5	20.1	22.9	0.7	400.4	<0.001
male	LFD	young	W5	26.5	25.0	28.0	0.8	464.1	
female	LFD	young	W6	21.8	20.3	23.2	0.7	400.4	<0.001
male	LFD	young	W6	26.8	25.2	28.3	0.8	464.1	
female	LFD	young	W7	21.7	20.3	23.1	0.7	400.4	<0.001
male	LFD	young	W7	27.0	25.5	28.6	0.8	464.1	
female	LFD	young	W8	22.7	21.0	24.4	0.9	616.8	<0.001
male	LFD	young	W8	27.2	25.2	29.1	1.0	781.3	

Supplementary Table 3. Comparisons by age for body weight.

age	diet	sex	timepoint	lsmean	lower.CL	upper.CL	SE	df	p.value
old	HFD	female	W0	30.3	28.6	32.0	0.9	581.6	<0.001
young	HFD	female	W0	19.5	18.0	21.0	0.8	440.0	
old	HFD	female	W1	30.8	28.7	33.0	1.1	873.4	<0.001
young	HFD	female	W1	19.9	18.5	21.4	0.8	440.0	
old	HFD	female	W2	31.4	29.2	33.7	1.2	919.6	<0.001
young	HFD	female	W2	21.7	20.2	23.2	0.8	440.0	

old	HFD	female	W3	33.6	31.4	35.8	1.1	873.4	<0.001
young	HFD	female	W3	22.4	20.9	23.9	0.8	440.0	
old	HFD	female	W4	35.6	34.0	37.3	0.9	581.6	<0.001
young	HFD	female	W4	23.2	21.7	24.7	0.8	440.0	
old	HFD	female	W5	35.3	33.6	37.0	0.9	616.8	<0.001
young	HFD	female	W5	23.5	22.0	25.0	0.8	440.0	
old	HFD	female	W6	36.6	34.9	38.4	0.9	616.8	<0.001
young	HFD	female	W6	25.3	23.8	26.8	0.8	440.0	
old	HFD	female	W7	36.9	35.2	38.6	0.9	616.8	<0.001
young	HFD	female	W7	25.2	23.7	26.7	0.8	440.0	
old	HFD	female	W8	37.7	36.0	39.4	0.9	581.6	<0.001
young	HFD	female	W8	26.7	24.9	28.6	0.9	695.1	
old	LFD	female	W0	30.0	28.1	31.9	1.0	736.8	<0.001
young	LFD	female	W0	19.7	18.2	21.1	0.7	400.4	
old	LFD	female	W1	29.2	27.0	31.4	1.1	873.3	<0.001
young	LFD	female	W1	20.3	18.9	21.7	0.7	400.4	
old	LFD	female	W2	29.8	27.7	32.0	1.1	873.3	<0.001
young	LFD	female	W2	20.6	19.2	22.0	0.7	400.4	
old	LFD	female	W3	29.4	26.8	32.0	1.3	1005.6	<0.001
young	LFD	female	W3	21.0	19.6	22.5	0.7	400.4	
old	LFD	female	W4	30.5	28.3	32.6	1.1	873.3	<0.001
young	LFD	female	W4	21.2	19.8	22.7	0.7	400.4	
old	LFD	female	W5	31.0	28.8	33.1	1.1	873.3	<0.001
young	LFD	female	W5	21.5	20.1	22.9	0.7	400.4	
old	LFD	female	W6	31.3	29.2	33.5	1.1	873.3	<0.001
young	LFD	female	W6	21.8	20.3	23.2	0.7	400.4	
old	LFD	female	W7	31.2	28.8	33.6	1.2	964.2	<0.001
young	LFD	female	W7	21.7	20.3	23.1	0.7	400.4	
old	LFD	female	W8	30.7	28.8	32.6	1.0	736.8	<0.001
young	LFD	female	W8	22.7	21.0	24.4	0.9	616.8	
old	HFD	male	W0	36.8	35.3	38.3	0.8	440.0	<0.001
young	HFD	male	W0	24.0	22.4	25.6	0.8	548.8	
old	HFD	male	W1	37.4	35.4	39.3	1.0	781.0	<0.001
young	HFD	male	W1	25.9	24.2	27.5	0.8	548.8	
old	HFD	male	W2	39.0	37.1	41.0	1.0	781.0	<0.001
young	HFD	male	W2	27.2	25.6	28.9	0.8	548.8	
old	HFD	male	W3	40.7	38.8	42.7	1.0	781.0	<0.001
young	HFD	male	W3	28.4	26.7	30.0	0.8	548.8	
old	HFD	male	W4	44.3	42.8	45.8	0.8	440.0	<0.001
young	HFD	male	W4	30.2	28.6	31.9	0.8	548.8	
old	HFD	male	W5	45.2	43.7	46.7	0.8	440.0	<0.001
young	HFD	male	W5	30.9	29.2	32.5	0.8	548.8	
old	HFD	male	W6	46.2	44.7	47.7	0.8	440.0	<0.001
young	HFD	male	W6	33.4	31.8	35.1	0.8	548.8	
old	HFD	male	W7	46.8	45.3	48.3	0.8	464.3	<0.001
young	HFD	male	W7	34.0	32.4	35.6	0.8	548.8	
old	HFD	male	W8	47.4	45.8	48.9	0.8	490.5	<0.001
young	HFD	male	W8	34.5	32.4	36.6	1.0	826.9	

old	LFD	male	W0	35.2	33.5	36.8	0.9	581.6	<0.001
young	LFD	male	W0	23.4	21.8	24.9	0.8	464.1	
old	LFD	male	W1	33.9	31.8	35.9	1.0	826.8	<0.001
young	LFD	male	W1	24.4	22.9	25.9	0.8	464.1	
old	LFD	male	W2	33.7	31.7	35.8	1.0	826.8	<0.001
young	LFD	male	W2	25.0	23.4	26.5	0.8	464.1	
old	LFD	male	W3	34.0	31.9	36.1	1.0	826.8	<0.001
young	LFD	male	W3	25.7	24.2	27.2	0.8	464.1	
old	LFD	male	W4	34.3	32.6	36.0	0.9	581.6	<0.001
young	LFD	male	W4	26.0	24.5	27.5	0.8	464.1	
old	LFD	male	W5	34.5	32.6	36.4	1.0	736.8	<0.001
young	LFD	male	W5	26.5	25.0	28.0	0.8	464.1	
old	LFD	male	W6	34.6	32.7	36.4	1.0	736.8	<0.001
young	LFD	male	W6	26.8	25.2	28.3	0.8	464.1	
old	LFD	male	W7	34.3	32.6	35.9	0.9	581.6	<0.001
young	LFD	male	W7	27.0	25.5	28.6	0.8	464.1	
old	LFD	male	W8	34.6	32.9	36.3	0.9	581.6	<0.001
young	LFD	male	W8	27.2	25.2	29.1	1.0	781.3	

Statistically significant differences for fat mass by diet, sex and age

Supplementary Table 4. Comparisons by diet for fat mass.

diet	age	sex	timepoint	lsmean	lower.CL	upper.CL	SE	df	p.value
HFD	old	female	W3	10.3	9.0	11.7	0.7	1024.1	0.014
LFD	old	female	W3	7.9	6.4	9.4	0.8	1049.8	
HFD	old	female	W4	11.9	10.9	13.0	0.5	893.9	<0.001
LFD	old	female	W4	8.1	6.8	9.4	0.7	1024.1	
HFD	old	female	W5	12.1	11.0	13.1	0.5	915.0	<0.001
LFD	old	female	W5	8.2	6.9	9.5	0.7	1024.1	
HFD	old	female	W6	12.8	11.7	13.8	0.5	915.0	<0.001
LFD	old	female	W6	8.7	7.4	10.0	0.7	1024.1	
HFD	old	female	W7	13.3	12.3	14.4	0.5	915.0	<0.001
LFD	old	female	W7	8.7	7.4	10.0	0.7	1024.1	
HFD	old	female	W8	13.6	12.6	14.7	0.5	893.9	<0.001
LFD	old	female	W8	8.1	6.7	9.5	0.7	1037.9	
HFD	old	male	W1	9.7	8.4	11.0	0.6	1008.9	<0.001
LFD	old	male	W1	6.7	5.4	8.0	0.7	1024.1	
HFD	old	male	W2	11.4	10.2	12.7	0.6	1008.9	<0.001
LFD	old	male	W2	6.8	5.5	8.2	0.7	1024.1	
HFD	old	male	W3	12.6	11.4	13.9	0.6	1008.9	<0.001
LFD	old	male	W3	6.9	5.6	8.3	0.7	1024.1	
HFD	old	male	W4	14.8	13.8	15.8	0.5	893.9	<0.001
LFD	old	male	W4	7.3	6.2	8.3	0.5	914.5	
HFD	old	male	W5	15.5	14.4	16.5	0.5	893.9	<0.001
LFD	old	male	W5	7.6	6.4	8.8	0.6	992.3	
HFD	old	male	W6	15.7	14.7	16.7	0.5	893.9	<0.001
LFD	old	male	W6	7.7	6.5	9.0	0.6	992.3	

HFD	old	male	W7	16.5	15.5	17.6	0.5	914.9	<0.001
LFD	old	male	W7	7.5	6.4	8.6	0.5	914.5	
HFD	old	male	W8	16.6	15.5	17.7	0.6	935.7	<0.001
LFD	old	male	W8	7.7	6.7	8.8	0.5	914.5	
HFD	young	female	W6	5.9	5.0	6.8	0.5	817.8	0.003
LFD	young	female	W6	4.1	3.2	4.9	0.4	757.5	
HFD	young	female	W7	6.0	5.1	6.9	0.5	817.8	0.002
LFD	young	female	W7	4.0	3.2	4.9	0.4	777.3	
HFD	young	female	W8	7.1	5.9	8.2	0.6	977.8	<0.001
LFD	young	female	W8	4.5	3.5	5.5	0.5	911.3	
HFD	young	male	W4	6.8	5.8	7.8	0.5	874.0	0.003
LFD	young	male	W4	4.7	3.7	5.8	0.5	914.5	
HFD	young	male	W5	7.5	6.5	8.5	0.5	874.0	<0.001
LFD	young	male	W5	4.8	3.7	5.9	0.5	914.5	
HFD	young	male	W6	8.8	7.8	9.8	0.5	874.0	<0.001
LFD	young	male	W6	4.9	3.9	6.0	0.5	914.5	
HFD	young	male	W7	9.4	8.4	10.4	0.5	893.9	<0.001
LFD	young	male	W7	4.9	3.9	6.0	0.5	914.5	
HFD	young	male	W8	9.5	8.3	10.8	0.6	1009.6	<0.001
LFD	young	male	W8	4.9	3.3	6.5	0.8	1059.4	

Supplementary Table 5. Comparisons by sex for fat mass.

sex	diet	age	timepoint	lsmean	lower.CL	upper.CL	SE	df	p.value
female	HFD	old	W2	8.9	7.5	10.3	0.7	1037.9	0.006
male	HFD	old	W2	11.4	10.2	12.7	0.6	1008.9	
female	HFD	old	W3	10.3	9.0	11.7	0.7	1024.1	0.011
male	HFD	old	W3	12.6	11.4	13.9	0.6	1008.9	
female	HFD	old	W4	11.9	10.9	13.0	0.5	893.9	<0.001
male	HFD	old	W4	14.8	13.8	15.8	0.5	893.9	
female	HFD	old	W5	12.1	11.0	13.1	0.5	915.0	<0.001
male	HFD	old	W5	15.5	14.4	16.5	0.5	893.9	
female	HFD	old	W6	12.8	11.7	13.8	0.5	915.0	<0.001
male	HFD	old	W6	15.7	14.7	16.7	0.5	893.9	
female	HFD	old	W7	13.3	12.3	14.4	0.5	915.0	<0.001
male	HFD	old	W7	16.5	15.5	17.6	0.5	914.9	
female	HFD	old	W8	13.6	12.6	14.7	0.5	893.9	<0.001
male	HFD	old	W8	16.6	15.5	17.7	0.6	935.7	
female	HFD	young	W3	4.5	3.6	5.4	0.5	817.8	0.032
male	HFD	young	W3	5.9	4.9	6.9	0.5	874.0	
female	HFD	young	W4	4.8	3.9	5.8	0.5	817.8	0.003
male	HFD	young	W4	6.8	5.8	7.8	0.5	874.0	
female	HFD	young	W5	4.9	3.9	5.8	0.5	838.6	<0.001
male	HFD	young	W5	7.5	6.5	8.5	0.5	874.0	
female	HFD	young	W6	5.9	5.0	6.8	0.5	817.8	<0.001
male	HFD	young	W6	8.8	7.8	9.8	0.5	874.0	
female	HFD	young	W7	6.0	5.1	6.9	0.5	817.8	<0.001
male	HFD	young	W7	9.4	8.4	10.4	0.5	893.9	

female	HFD	young	W8	7.1	5.9	8.2	0.6	977.8	0.004
male	HFD	young	W8	9.5	8.3	10.8	0.6	1009.6	

Supplementary Table 6. Comparisons by age for fat mass.

age	diet	sex	timepoint	lsmean	lower.CL	upper.CL	SE	df	p.value
old	HFD	female	W0	8.2	7.2	9.2	0.5	893.9	<0.001
young	HFD	female	W0	3.5	2.5	4.4	0.5	817.8	
old	HFD	female	W1	8.1	6.7	9.4	0.7	1024.1	<0.001
young	HFD	female	W1	3.6	2.7	4.5	0.5	817.8	
old	HFD	female	W2	8.9	7.5	10.3	0.7	1037.9	<0.001
young	HFD	female	W2	4.3	3.4	5.3	0.5	817.8	
old	HFD	female	W3	10.3	9.0	11.7	0.7	1024.1	<0.001
young	HFD	female	W3	4.5	3.6	5.4	0.5	817.8	
old	HFD	female	W4	11.9	10.9	13.0	0.5	893.9	<0.001
young	HFD	female	W4	4.8	3.9	5.8	0.5	817.8	
old	HFD	female	W5	12.1	11.0	13.1	0.5	915.0	<0.001
young	HFD	female	W5	4.9	3.9	5.8	0.5	838.6	
old	HFD	female	W6	12.8	11.7	13.8	0.5	915.0	<0.001
young	HFD	female	W6	5.9	5.0	6.8	0.5	817.8	
old	HFD	female	W7	13.3	12.3	14.4	0.5	915.0	<0.001
young	HFD	female	W7	6.0	5.1	6.9	0.5	817.8	
old	HFD	female	W8	13.6	12.6	14.7	0.5	893.9	<0.001
young	HFD	female	W8	7.1	5.9	8.2	0.6	977.8	
old	LFD	female	W0	8.3	7.0	9.7	0.7	1024.1	<0.001
young	LFD	female	W0	3.5	2.6	4.3	0.4	757.5	
old	LFD	female	W1	8.0	6.5	9.5	0.8	1049.8	<0.001
young	LFD	female	W1	3.7	2.9	4.6	0.4	757.5	
old	LFD	female	W2	8.1	6.6	9.6	0.8	1049.8	<0.001
young	LFD	female	W2	3.9	3.0	4.7	0.4	757.5	
old	LFD	female	W3	7.9	6.4	9.4	0.8	1049.8	<0.001
young	LFD	female	W3	4.0	3.2	4.9	0.4	757.5	
old	LFD	female	W4	8.1	6.8	9.4	0.7	1024.1	<0.001
young	LFD	female	W4	3.9	3.0	4.7	0.4	757.5	
old	LFD	female	W5	8.2	6.9	9.5	0.7	1024.1	<0.001
young	LFD	female	W5	4.0	3.1	4.9	0.4	757.5	
old	LFD	female	W6	8.7	7.4	10.0	0.7	1024.1	<0.001
young	LFD	female	W6	4.1	3.2	4.9	0.4	757.5	
old	LFD	female	W7	8.7	7.4	10.0	0.7	1024.1	<0.001
young	LFD	female	W7	4.0	3.2	4.9	0.4	777.3	
old	LFD	female	W8	8.1	6.7	9.5	0.7	1037.9	<0.001
young	LFD	female	W8	4.5	3.5	5.5	0.5	911.3	
old	HFD	male	W0	8.4	7.4	9.4	0.5	893.9	<0.001
young	HFD	male	W0	4.0	3.0	5.0	0.5	874.0	
old	HFD	male	W1	9.7	8.4	11.0	0.6	1008.9	<0.001
young	HFD	male	W1	4.7	3.7	5.7	0.5	874.0	
old	HFD	male	W2	11.4	10.2	12.7	0.6	1008.9	<0.001
young	HFD	male	W2	5.4	4.4	6.4	0.5	874.0	

old	HFD	male	W3	12.6	11.4	13.9	0.6	1008.9	<0.001
young	HFD	male	W3	5.9	4.9	6.9	0.5	874.0	
old	HFD	male	W4	14.8	13.8	15.8	0.5	893.9	<0.001
young	HFD	male	W4	6.8	5.8	7.8	0.5	874.0	
old	HFD	male	W5	15.5	14.4	16.5	0.5	893.9	<0.001
young	HFD	male	W5	7.5	6.5	8.5	0.5	874.0	
old	HFD	male	W6	15.7	14.7	16.7	0.5	893.9	<0.001
young	HFD	male	W6	8.8	7.8	9.8	0.5	874.0	
old	HFD	male	W7	16.5	15.5	17.6	0.5	914.9	<0.001
young	HFD	male	W7	9.4	8.4	10.4	0.5	893.9	
old	HFD	male	W8	16.6	15.5	17.7	0.6	935.7	<0.001
young	HFD	male	W8	9.5	8.3	10.8	0.6	1009.6	
old	LFD	male	W0	8.0	6.9	9.1	0.5	914.5	<0.001
young	LFD	male	W0	3.9	2.9	5.0	0.5	914.5	
old	LFD	male	W1	6.7	5.4	8.0	0.7	1024.1	0.005
young	LFD	male	W1	4.3	3.3	5.4	0.5	914.5	
old	LFD	male	W2	6.8	5.5	8.2	0.7	1024.1	0.003
young	LFD	male	W2	4.4	3.3	5.4	0.5	914.5	
old	LFD	male	W3	6.9	5.6	8.3	0.7	1024.1	0.008
young	LFD	male	W3	4.7	3.6	5.8	0.5	914.5	
old	LFD	male	W4	7.3	6.2	8.3	0.5	914.5	<0.001
young	LFD	male	W4	4.7	3.7	5.8	0.5	914.5	
old	LFD	male	W5	7.6	6.4	8.8	0.6	992.3	<0.001
young	LFD	male	W5	4.8	3.7	5.9	0.5	914.5	
old	LFD	male	W6	7.7	6.5	9.0	0.6	992.3	<0.001
young	LFD	male	W6	4.9	3.9	6.0	0.5	914.5	
old	LFD	male	W7	7.5	6.4	8.6	0.5	914.5	<0.001
young	LFD	male	W7	4.9	3.9	6.0	0.5	914.5	
old	LFD	male	W8	7.7	6.7	8.8	0.5	914.5	0.003

Statistically significant differences for lean mass by diet, sex and age

Supplementary Table 7. Comparisons by diet for lean mass.

diet	age	sex	timepoint	lsmean	lower.CL	upper.CL	SE	df	p.value
HFD	old	male	W5	26.8	26.0	27.7	0.5	566.9	0.018
LFD	old	male	W5	25.3	24.3	26.3	0.5	747.6	
HFD	old	male	W7	27.2	26.3	28.2	0.5	599.0	0.002
LFD	old	male	W7	25.4	24.5	26.3	0.5	598.9	
HFD	old	male	W8	26.9	26.0	27.8	0.5	633.1	<0.001
LFD	old	male	W8	24.8	23.9	25.7	0.5	598.9	
HFD	young	male	W6	22.3	21.5	23.2	0.4	537.1	0.007
LFD	young	male	W6	20.8	19.9	21.7	0.5	566.9	
HFD	young	male	W7	22.6	21.7	23.5	0.5	566.9	0.010
LFD	young	male	W7	21.1	20.2	22.0	0.5	566.9	
HFD	young	male	W8	23.2	22.1	24.2	0.5	789.3	0.003

Supplementary Table 8. Comparisons by sex for lean mass.

sex	diet	age	timepoint	lsmean	lower.CL	upper.CL	SE	df	p.value
female	HFD	old	W0	20.8	19.9	21.7	0.5	566.9	<0.001
male	HFD	old	W0	25.0	24.2	25.9	0.5	566.9	
female	HFD	old	W1	20.7	19.6	21.9	0.6	831.6	<0.001
male	HFD	old	W1	24.9	23.9	26.0	0.5	789.3	
female	HFD	old	W2	21.1	19.9	22.3	0.6	874.1	<0.001
male	HFD	old	W2	25.2	24.1	26.3	0.5	789.3	
female	HFD	old	W3	22.0	20.9	23.1	0.6	831.6	<0.001
male	HFD	old	W3	25.7	24.6	26.7	0.5	789.3	
female	HFD	old	W4	22.4	21.5	23.3	0.5	566.9	<0.001
male	HFD	old	W4	26.6	25.7	27.5	0.5	566.9	
female	HFD	old	W5	21.6	20.7	22.5	0.5	598.9	<0.001
male	HFD	old	W5	26.8	26.0	27.7	0.5	566.9	
female	HFD	old	W6	22.2	21.3	23.1	0.5	598.9	<0.001
male	HFD	old	W6	27.1	26.3	28.0	0.5	566.9	
female	HFD	old	W7	22.3	21.4	23.2	0.5	598.9	<0.001
male	HFD	old	W7	27.2	26.3	28.2	0.5	599.0	
female	HFD	old	W8	22.3	21.5	23.2	0.5	566.9	<0.001
male	HFD	old	W8	26.9	26.0	27.8	0.5	633.1	
female	LFD	old	W0	21.0	19.7	22.3	0.7	955.6	<0.001
male	LFD	old	W0	25.7	24.8	26.7	0.5	598.9	
female	LFD	old	W1	21.7	20.5	22.8	0.6	874.1	<0.001
male	LFD	old	W1	25.9	24.8	27.0	0.6	831.6	
female	LFD	old	W2	21.1	19.7	22.6	0.7	991.8	<0.001
male	LFD	old	W2	24.6	23.2	26.1	0.7	991.8	
female	LFD	old	W3	20.9	19.5	22.4	0.7	991.8	<0.001
male	LFD	old	W3	25.5	24.4	26.7	0.6	831.6	
female	LFD	old	W4	21.6	20.5	22.8	0.6	874.1	<0.001
male	LFD	old	W4	25.8	24.9	26.7	0.5	598.9	
female	LFD	old	W5	21.3	20.2	22.5	0.6	874.1	<0.001
male	LFD	old	W5	25.3	24.3	26.3	0.5	747.6	
female	LFD	old	W6	21.6	20.4	22.8	0.6	874.1	<0.001
male	LFD	old	W6	26.0	24.9	27.0	0.5	747.6	
female	LFD	old	W7	21.4	20.1	22.8	0.7	955.6	<0.001
male	LFD	old	W7	25.4	24.5	26.3	0.5	598.9	
female	LFD	old	W8	21.2	20.0	22.5	0.6	915.9	<0.001
male	LFD	old	W8	24.8	23.9	25.7	0.5	598.9	
female	HFD	young	W0	15.6	14.7	16.4	0.4	460.2	<0.001
male	HFD	young	W0	18.8	17.9	19.6	0.4	537.1	
female	HFD	young	W1	15.1	14.3	15.9	0.4	438.9	<0.001
male	HFD	young	W1	19.3	18.4	20.2	0.4	537.1	
female	HFD	young	W2	16.5	15.7	17.3	0.4	460.2	<0.001
male	HFD	young	W2	19.9	19.0	20.7	0.4	537.1	
female	HFD	young	W3	17.0	16.2	17.8	0.4	460.2	<0.001
male	HFD	young	W3	20.5	19.6	21.3	0.4	537.1	
female	HFD	young	W4	17.3	16.5	18.1	0.4	460.2	<0.001
male	HFD	young	W4	21.5	20.6	22.3	0.4	537.1	

female	HFD	young	W5	17.4	16.6	18.2	0.4	484.5	<0.001
male	HFD	young	W5	21.6	20.7	22.5	0.4	537.1	
female	HFD	young	W6	18.1	17.3	18.9	0.4	460.2	<0.001
male	HFD	young	W6	22.3	21.5	23.2	0.4	537.1	
female	HFD	young	W7	18.2	17.4	19.0	0.4	460.2	<0.001
male	HFD	young	W7	22.6	21.7	23.5	0.5	566.9	
female	HFD	young	W8	18.6	17.6	19.6	0.5	709.0	<0.001
male	HFD	young	W8	23.2	22.1	24.2	0.5	789.3	
female	LFD	young	W0	15.9	15.2	16.7	0.4	383.4	<0.001
male	LFD	young	W0	18.5	17.6	19.4	0.5	566.9	
female	LFD	young	W1	15.9	15.2	16.7	0.4	383.4	<0.001
male	LFD	young	W1	18.9	18.0	19.8	0.5	566.9	
female	LFD	young	W2	16.4	15.6	17.1	0.4	383.4	<0.001
male	LFD	young	W2	19.6	18.7	20.5	0.5	566.9	
female	LFD	young	W3	16.5	15.8	17.3	0.4	383.4	<0.001
male	LFD	young	W3	19.9	19.0	20.8	0.5	566.9	
female	LFD	young	W4	16.8	16.0	17.5	0.4	383.4	<0.001
male	LFD	young	W4	20.4	19.5	21.3	0.5	566.9	
female	LFD	young	W5	17.0	16.2	17.7	0.4	383.4	<0.001
male	LFD	young	W5	20.5	19.6	21.4	0.5	566.9	
female	LFD	young	W6	17.1	16.4	17.9	0.4	383.4	<0.001
male	LFD	young	W6	20.8	19.9	21.7	0.5	566.9	
female	LFD	young	W7	17.3	16.5	18.0	0.4	383.4	<0.001
male	LFD	young	W7	21.1	20.2	22.0	0.5	566.9	
female	LFD	young	W8	17.6	16.8	18.5	0.5	573.4	<0.001
male	LFD	young	W8	20.8	19.6	22.1	0.6	915.8	

Supplementary Table 9. Comparisons by age for lean mass.

age	diet	sex	timepoint	lsmean	lower.CL	upper.CL	SE	df	p.value
old	HFD	female	W0	20.8	19.9	21.7	0.5	566.9	<0.001
young	HFD	female	W0	15.6	14.7	16.4	0.4	460.2	
old	HFD	female	W1	20.7	19.6	21.9	0.6	831.6	<0.001
young	HFD	female	W1	15.1	14.3	15.9	0.4	438.9	
old	HFD	female	W2	21.1	19.9	22.3	0.6	874.1	<0.001
young	HFD	female	W2	16.5	15.7	17.3	0.4	460.2	
old	HFD	female	W3	22.0	20.9	23.1	0.6	831.6	<0.001
young	HFD	female	W3	17.0	16.2	17.8	0.4	460.2	
old	HFD	female	W4	22.4	21.5	23.3	0.5	566.9	<0.001
young	HFD	female	W4	17.3	16.5	18.1	0.4	460.2	
old	HFD	female	W5	21.6	20.7	22.5	0.5	598.9	<0.001
young	HFD	female	W5	17.4	16.6	18.2	0.4	484.5	
old	HFD	female	W6	22.2	21.3	23.1	0.5	598.9	<0.001
young	HFD	female	W6	18.1	17.3	18.9	0.4	460.2	
old	HFD	female	W7	22.3	21.4	23.2	0.5	598.9	<0.001
young	HFD	female	W7	18.2	17.4	19.0	0.4	460.2	
old	HFD	female	W8	22.3	21.5	23.2	0.5	566.9	<0.001
young	HFD	female	W8	18.6	17.6	19.6	0.5	709.0	

old	LFD	female	W0	21.0	19.7	22.3	0.7	955.6	<0.001
young	LFD	female	W0	15.9	15.2	16.7	0.4	383.4	
old	LFD	female	W1	21.7	20.5	22.8	0.6	874.1	<0.001
young	LFD	female	W1	15.9	15.2	16.7	0.4	383.4	
old	LFD	female	W2	21.1	19.7	22.6	0.7	991.8	<0.001
young	LFD	female	W2	16.4	15.6	17.1	0.4	383.4	
old	LFD	female	W3	20.9	19.5	22.4	0.7	991.8	<0.001
young	LFD	female	W3	16.5	15.8	17.3	0.4	383.4	
old	LFD	female	W4	21.6	20.5	22.8	0.6	874.1	<0.001
young	LFD	female	W4	16.8	16.0	17.5	0.4	383.4	
old	LFD	female	W5	21.3	20.2	22.5	0.6	874.1	<0.001
young	LFD	female	W5	17.0	16.2	17.7	0.4	383.4	
old	LFD	female	W6	21.6	20.4	22.8	0.6	874.1	<0.001
young	LFD	female	W6	17.1	16.4	17.9	0.4	383.4	
old	LFD	female	W7	21.4	20.1	22.8	0.7	955.6	<0.001
young	LFD	female	W7	17.3	16.5	18.0	0.4	383.4	
old	LFD	female	W8	21.2	20.0	22.5	0.6	915.9	<0.001
young	LFD	female	W8	17.6	16.8	18.5	0.5	573.4	
old	HFD	male	W0	25.0	24.2	25.9	0.5	566.9	<0.001
young	HFD	male	W0	18.8	17.9	19.6	0.4	537.1	
old	HFD	male	W1	24.9	23.9	26.0	0.5	789.3	<0.001
young	HFD	male	W1	19.3	18.4	20.2	0.4	537.1	
old	HFD	male	W2	25.2	24.1	26.3	0.5	789.3	<0.001
young	HFD	male	W2	19.9	19.0	20.7	0.4	537.1	
old	HFD	male	W3	25.7	24.6	26.7	0.5	789.3	<0.001
young	HFD	male	W3	20.5	19.6	21.3	0.4	537.1	
old	HFD	male	W4	26.6	25.7	27.5	0.5	566.9	<0.001
young	HFD	male	W4	21.5	20.6	22.3	0.4	537.1	
old	HFD	male	W5	26.8	26.0	27.7	0.5	566.9	<0.001
young	HFD	male	W5	21.6	20.7	22.5	0.4	537.1	
old	HFD	male	W6	27.1	26.3	28.0	0.5	566.9	<0.001
young	HFD	male	W6	22.3	21.5	23.2	0.4	537.1	
old	HFD	male	W7	27.2	26.3	28.2	0.5	599.0	<0.001
young	HFD	male	W7	22.6	21.7	23.5	0.5	566.9	
old	HFD	male	W8	26.9	26.0	27.8	0.5	633.1	<0.001
young	HFD	male	W8	23.2	22.1	24.2	0.5	789.3	
old	LFD	male	W0	25.7	24.8	26.7	0.5	598.9	<0.001
young	LFD	male	W0	18.5	17.6	19.4	0.5	566.9	
old	LFD	male	W1	25.9	24.8	27.0	0.6	831.6	<0.001
young	LFD	male	W1	18.9	18.0	19.8	0.5	566.9	
old	LFD	male	W2	24.6	23.2	26.1	0.7	991.8	<0.001
young	LFD	male	W2	19.6	18.7	20.5	0.5	566.9	
old	LFD	male	W3	25.5	24.4	26.7	0.6	831.6	<0.001
young	LFD	male	W3	19.9	19.0	20.8	0.5	566.9	
old	LFD	male	W4	25.8	24.9	26.7	0.5	598.9	<0.001
young	LFD	male	W4	20.4	19.5	21.3	0.5	566.9	
old	LFD	male	W5	25.3	24.3	26.3	0.5	747.6	<0.001
young	LFD	male	W5	20.5	19.6	21.4	0.5	566.9	

old	LFD	male	W6	26.0	24.9	27.0	0.5	747.6	<0.001
young	LFD	male	W6	20.8	19.9	21.7	0.5	566.9	
old	LFD	male	W7	25.4	24.5	26.3	0.5	598.9	<0.001
young	LFD	male	W7	21.1	20.2	22.0	0.5	566.9	
old	LFD	male	W8	24.8	23.9	25.7	0.5	598.9	<0.001
young	LFD	male	W8	20.8	19.6	22.1	0.6	915.8	

Statistically significant differences for dermis (fibroblast rich dermis +dWAT) by diet and age

Supplementary Table 10. Summary of coefficients of model for dermis (fibroblast rich dermis + dWAT): diet.

	Estimate	Std. Error	t value	Pr(>|t|)
(Intercept)	504	37.7	13.38	0.000
diet - LFD	-144	53.3	-2.71	0.013

Statistically significant differences for dWAT by diet and age

Supplementary Table 11. Comparisons by diet for dWAT.

diet	age	lsmean	lower.CL	upper.CL	SE	df	p.value
HFD	old	331.3	247.4	415.2	40.2	20.0	0.009
LFD	old	167.2	83.3	251.2	40.2	20.0	
HFD	young	291.5	207.6	375.4	40.2	20.0	0.049
LFD	young	172.4	88.5	256.3	40.2	20.0	

Supplementary Table 12. Summary of coefficients of model for dWAT: diet.

	Estimate	Std. Error	t value	Pr(>|t|)
(Intercept)	311	27.5	11.34	0.000
diet - LFD	-142	38.8	-3.65	0.001

Statistically significant differences for fibroblasts rich dermis by diet and age

Supplementary Table 13. Summary of coefficients of model for fibroblasts rich dermis: age.

	Estimate	Std. Error	t value	Pr(>|t|)
(Intercept)	153.9	23.9	6.43	0.000
age - young	74.9	33.8	2.21	0.038

Statistically significant differences for MCP-1 by diet and age

Supplementary Table 14. Comparisons by age for MCP-1.

age	diet	day	lsmean	lower.CL	upper.CL	SE	df	p.value
old	HFD	D3	271.5	214.8	328.2	28.3	60.0	0.002
young	HFD	D3	138.5	81.8	195.1	28.3	60.0	
old	LFD	D0	149.4	92.8	206.1	28.3	60.0	0.047
young	LFD	D0	68.2	11.6	124.9	28.3	60.0	

old	LFD	D3	266.3	209.6	322.9	28.3	60.0	0.001
young	LFD	D3	128.9	72.2	185.6	28.3	60.0	

Supplementary Table 15. Summary of coefficients of model for MCP-1: age + diet + day + age:day.

	Estimate	Std. Error	t value	Pr(>|t|)
(Intercept)	95.83	21.7	4.417	0.000
age - young	-40.02	28.4	-1.409	0.164
diet - LFD	29.73	16.4	1.813	0.074
dayD3	158.21	28.4	5.569	0.000
dayD7	28.20	28.4	0.993	0.325
age - young:dayD3	-95.19	40.2	-2.370	0.021
age - young:dayD7	-3.69	40.2	-0.092	0.927

Statistically significant differences for *CD68* mRNA expression by diet, sex and age

Supplementary Table 16. Comparisons by diet for *CD68* mRNA expression.

diet	age	sex	day	lsmean	lower.CL	upper.CL	SE	df	p.value
HFD	young	female	D0	2.4	0.8	4.0	0.8	192.0	<0.001
LFD	young	female	D0	6.2	4.7	7.8	0.8	192.0	
HFD	young	female	D7	9.8	7.7	11.8	1.0	192.0	0.027
LFD	young	female	D7	6.6	4.8	8.5	0.9	192.0	
HFD	young	male	D0	3.6	1.9	5.3	0.9	192.0	0.005
LFD	young	male	D0	7.0	5.4	8.7	0.9	192.0	
HFD	young	male	D3	9.1	7.3	11.0	0.9	192.0	0.023
LFD	young	male	D3	11.9	10.4	13.5	0.8	192.0	

Supplementary Table 17. Comparisons by sex for *CD68* mRNA expression.

sex	diet	age	day	lsmean	lower.CL	upper.CL	SE	df	p.value
female	LFD	old	D7	3.2	1.5	4.9	0.9	192.0	0.013
male	LFD	old	D7	6.3	4.6	8.0	0.9	192.0	
female	HFD	young	D3	12.6	10.8	14.5	0.9	192.0	0.009
male	HFD	young	D3	9.1	7.3	11.0	0.9	192.0	

Supplementary Table 18. Comparisons by age for *CD68* mRNA expression.

age	diet	sex	day	lsmean	lower.CL	upper.CL	SE	df	p.value
old	HFD	female	D3	8.9	7.2	10.6	0.9	192.0	0.004
young	HFD	female	D3	12.6	10.8	14.5	0.9	192.0	
old	HFD	female	D7	3.8	1.7	5.9	1.0	192.0	<0.001
young	HFD	female	D7	9.8	7.7	11.8	1.0	192.0	
old	LFD	female	D0	2.3	0.6	4.0	0.9	192.0	0.001
young	LFD	female	D0	6.2	4.7	7.8	0.8	192.0	
old	LFD	female	D7	3.2	1.5	4.9	0.9	192.0	0.008
young	LFD	female	D7	6.6	4.8	8.5	0.9	192.0	
old	LFD	female	D14	1.3	-0.4	3.0	0.9	192.0	0.002

young	LFD	female	D14	5.1	3.4	6.8	0.9	192.0	
old	LFD	female	D21	1.4	-0.3	3.1	0.9	192.0	0.035
young	LFD	female	D21	4.0	2.3	5.7	0.9	192.0	
old	HFD	male	D21	2.2	0.3	4.0	0.9	192.0	0.040
young	HFD	male	D21	4.8	3.1	6.5	0.9	192.0	
old	LFD	male	D0	2.9	1.4	4.5	0.8	192.0	<0.001
young	LFD	male	D0	7.0	5.4	8.7	0.9	192.0	
old	LFD	male	D3	8.5	6.9	10.2	0.9	192.0	0.004
young	LFD	male	D3	11.9	10.4	13.5	0.8	192.0	
old	LFD	male	D14	1.9	0.2	3.6	0.9	192.0	0.016
young	LFD	male	D14	5.0	3.1	6.8	0.9	192.0	
old	LFD	male	D21	1.6	-0.1	3.3	0.9	192.0	<0.001
young	LFD	male	D21	6.3	4.6	7.9	0.9	192.0	

Supplementary Table 19. Summary of coefficients of model for *CD68* mRNA expression: age + sex + diet + day + age:sex + age:diet + age:day + sex:day + diet:day + age:sex:day + age:diet:day.

	Estimate	Std. Error	t value	Pr(>|t|)
(Intercept)	1.099	0.658	1.671	0.096
age - young	1.377	0.958	1.437	0.152
sex - male	1.207	0.796	1.515	0.131
diet - LFD	0.904	0.799	1.133	0.259
dayD3	7.970	1.003	7.949	0.000
dayD7	2.227	1.081	2.059	0.041
dayD14	1.201	0.992	1.210	0.228
dayD21	0.536	0.997	0.538	0.591
age - young:sex - male	-0.205	1.148	-0.179	0.858
age - young:diet - LFD	2.761	1.148	2.405	0.017
age - young:dayD3	0.761	1.458	0.522	0.602
age - young:dayD7	4.501	1.560	2.885	0.004
age - young:dayD14	0.639	1.425	0.448	0.654
age - young:dayD21	-0.536	1.488	-0.360	0.719
sex - male:dayD3	-1.420	1.205	-1.178	0.240
sex - male:dayD7	1.193	1.205	0.990	0.324
sex - male:dayD14	-0.319	1.171	-0.273	0.785
sex - male:dayD21	-0.792	1.186	-0.668	0.505
diet - LFD:dayD3	-1.033	1.207	-0.856	0.393
diet - LFD:dayD7	-0.675	1.207	-0.560	0.576
diet - LFD:dayD14	-2.061	1.172	-1.758	0.080
diet - LFD:dayD21	-1.242	1.187	-1.046	0.297
age - young:sex - male:dayD3	-0.290	1.705	-0.170	0.865
age - young:sex - male:dayD7	-3.440	1.753	-1.963	0.051
age - young:sex - male:dayD14	-0.193	1.681	-0.115	0.909
age - young:sex - male:dayD21	2.098	1.705	1.230	0.220
age - young:diet - LFD:dayD3	-2.205	1.708	-1.291	0.198
age - young:diet - LFD:dayD7	-5.096	1.752	-2.908	0.004
age - young:diet - LFD:dayD14	-1.107	1.681	-0.659	0.511
age - young:diet - LFD:dayD21	-0.940	1.705	-0.552	0.582

Statistically significant differences for re-epithelialization by diet and age

Supplementary Table 20. Comparisons by diet for reepithelialization.

diet	age	day	lsmean	lower.CL	upper.CL	SE	df	p.value
HFD	young	D3	24.8	2.9	46.7	10.9	46.0	0.019
LFD	young	D3	63.4	40.3	86.4	11.5	46.0	

Supplementary Table 21. Comparisons by age for re-epithelialization.

age	diet	day	lsmean	lower.CL	upper.CL	SE	df	p.value
old	LFD	D3	25.9	-2.4	54.2	14.0	46.0	0.045
young	LFD	D3	63.4	40.3	86.4	11.5	46.0	

Supplementary Table 22. Summary of coefficients of model for re-epithelialization: age + diet + day + age:diet.

	Estimate	Std. Error	t value	Pr(>|t|)
(Intercept)	32.82	9.68	3.390	0.001
age - young	-2.91	11.96	-0.243	0.809
diet - LFD	-8.20	13.61	-0.602	0.550
dayD7	47.82	9.56	5.001	0.000
age - young:diet - LFD	38.85	18.70	2.078	0.043

Statistically significant differences for *Mmp-9* mRNA expression by diet, sex and age

Supplementary Table 23. Comparisons by diet for *Mmp-9* mRNA expression.

diet	age	sex	day	lsmean	lower.CL	upper.CL	SE	df	p.value
HFD	old	female	D3	42.6	35.8	49.4	3.5	197.0	0.003
LFD	old	female	D3	27.1	19.6	34.5	3.8	197.0	
HFD	old	male	D7	11.5	4.7	18.3	3.5	197.0	0.008
LFD	old	male	D7	24.5	17.7	31.3	3.5	197.0	

Supplementary Table 24. Comparisons by sex for *Mmp-9* mRNA expression.

sex	diet	age	day	lsmean	lower.CL	upper.CL	SE	df	p.value
female	LFD	old	D3	27.1	19.6	34.5	3.8	197.0	0.001
male	LFD	old	D3	43.7	36.9	50.5	3.5	197.0	
female	HFD	young	D3	22.3	15.5	29.1	3.5	197.0	0.014
male	HFD	young	D3	35.0	27.5	42.5	3.8	197.0	

Supplementary Table 25. Comparisons by age for *Mmp-9* mRNA expression.

age	diet	sex	day	lsmean	lower.CL	upper.CL	SE	df	p.value
old	HFD	female	D3	42.6	35.8	49.4	3.5	197.0	<0.001
young	HFD	female	D3	22.3	15.5	29.1	3.5	197.0	
old	HFD	male	D3	45.9	39.1	52.7	3.5	197.0	0.034
young	HFD	male	D3	35.0	27.5	42.5	3.8	197.0	

old	LFD	male	D3	43.7	36.9	50.5	3.5	197.0	<0.001
young	LFD	male	D3	25.5	19.2	31.8	3.2	197.0	

Supplementary Table 26. Summary of coefficients of model for *Mmp-9* mRNA expression: age + sex + diet + day + age:day + diet:day.

	Estimate	Std. Error	t value	Pr(>|t|)
(Intercept)	4.202	2.00	2.100	0.037
age - young	1.238	2.31	0.536	0.593
sex - male	1.801	1.10	1.631	0.104
diet - LFD	4.696	2.31	2.032	0.043
dayD3	37.676	2.87	13.117	0.000
dayD7	10.926	2.99	3.653	0.000
dayD14	0.197	2.88	0.069	0.945
dayD21	-0.703	2.92	-0.240	0.810
age - young:dayD3	-14.272	3.39	-4.211	0.000
age - young:dayD7	-3.215	3.45	-0.932	0.352
age - young:dayD14	0.238	3.41	0.070	0.944
age - young:dayD21	-0.890	3.41	-0.261	0.794
diet - LFD:dayD3	-9.791	3.39	-2.889	0.004
diet - LFD:dayD7	-0.292	3.45	-0.084	0.933
diet - LFD:dayD14	-4.087	3.41	-1.200	0.231
diet - LFD:dayD21	-3.718	3.41	-1.091	0.276

Statistically significant differences for *Timp-1* mRNA expression by diet, sex and age

Supplementary Table 27. Comparisons by diet for *Timp-1* mRNA expression.

diet	age	sex	day	lsmean	lower.CL	upper.CL	SE	df	p.value
HFD	old	female	D3	157.2	136.8	177.6	10.3	197.0	<0.001
LFD	old	female	D3	44.4	22.1	66.8	11.3	197.0	
HFD	young	female	D3	82.4	62.0	102.8	10.3	197.0	0.011
LFD	young	female	D3	44.9	24.5	65.3	10.3	197.0	
HFD	young	female	D7	57.5	35.2	79.9	11.3	197.0	0.019
LFD	young	female	D7	19.5	-2.9	41.8	11.3	197.0	
HFD	young	male	D3	76.7	54.4	99.1	11.3	197.0	0.042
LFD	young	male	D3	46.4	27.5	65.3	9.6	197.0	

Supplementary Table 28. Comparisons by sex for *Timp-1* mRNA expression.

sex	diet	age	day	lsmean	lower.CL	upper.CL	SE	df	p.value
female	HFD	old	D3	157.2	136.8	177.6	10.3	197.0	<0.001
male	HFD	old	D3	88.0	67.6	108.4	10.3	197.0	

Supplementary Table 29. Comparisons by age for *Timp-1* mRNA expression.

age	diet	sex	day	lsmean	lower.CL	upper.CL	SE	df	p.value
old	HFD	female	D3	157.2	136.8	177.6	10.3	197.0	<0.001

young	HFD	female	D3	82.4	62.0	102.8	10.3	197.0

Supplementary Table 30. Summary of coefficients of model for *Timp-1* mRNA expression: age * sex * diet * day.

	Estimate	Std. Error	t value	Pr(>|t|)
(Intercept)	1.993	8.96	0.222	0.824
age - young	0.380	13.12	0.029	0.977
sex - male	0.239	13.12	0.018	0.985
diet - LFD	3.203	13.69	0.234	0.815
dayD3	155.186	13.69	11.335	0.000
dayD7	30.090	15.52	1.938	0.054
dayD14	4.785	13.69	0.350	0.727
dayD21	7.882	13.69	0.576	0.565
age - young:sex - male	0.560	19.26	0.029	0.977
age - young:diet - LFD	-1.877	19.26	-0.097	0.922
sex - male:diet - LFD	-4.006	19.26	-0.208	0.835
age - young:dayD3	-75.128	19.66	-3.822	0.000
age - young:dayD7	25.045	21.48	1.166	0.245
age - young:dayD14	3.341	20.19	0.165	0.869
age - young:dayD21	-8.121	20.19	-0.402	0.688
sex - male:dayD3	-69.406	19.66	-3.531	0.001
sex - male:dayD7	-22.419	20.97	-1.069	0.286
sex - male:dayD14	5.978	19.66	0.304	0.761
sex - male:dayD21	-8.068	20.19	-0.400	0.690
diet - LFD:dayD3	-115.937	20.57	-5.637	0.000
diet - LFD:dayD7	11.831	21.34	0.555	0.580
diet - LFD:dayD14	-6.906	20.04	-0.345	0.731
diet - LFD:dayD21	-8.225	20.04	-0.410	0.682
age - young:sex - male:diet - LFD	9.248	27.73	0.334	0.739
age - young:sex - male:dayD3	62.913	28.65	2.196	0.029
age - young:sex - male:dayD7	1.109	29.57	0.037	0.970
age - young:sex - male:dayD14	-8.604	28.65	-0.300	0.764
age - young:sex - male:dayD21	10.156	29.02	0.350	0.727
age - young:diet - LFD:dayD3	77.065	28.65	2.690	0.008
age - young:diet - LFD:dayD7	-51.200	29.93	-1.711	0.089
age - young:diet - LFD:dayD14	0.732	28.65	0.026	0.980
age - young:diet - LFD:dayD21	8.596	28.65	0.300	0.764
sex - male:diet - LFD:dayD3	97.531	28.65	3.404	0.001
sex - male:diet - LFD:dayD7	13.657	29.21	0.468	0.641
sex - male:diet - LFD:dayD14	-0.840	28.27	-0.030	0.976
sex - male:diet - LFD:dayD21	8.937	28.65	0.312	0.755
age - young:sex - male:diet - LFD:dayD3	-95.603	40.66	-2.351	0.020
age - young:sex - male:diet - LFD:dayD7	26.887	41.50	0.648	0.518
age - young:sex - male:diet - LFD:dayD14	0.889	40.85	0.022	0.983
age - young:sex - male:diet - LFD:dayD21	-8.808	40.85	-0.216	0.829

Statistically significant differences for *Mmp-13* mRNA expression by diet, sex and age

Supplementary Table 31. Comparisons by diet for *Mmp-13* mRNA expression.

diet	age	sex	day	lsmean	lower.CL	upper.CL	SE	df	p.value
HFD	young	female	D7	228.5	181.0	276.0	24.1	193.0	<0.001
LFD	young	female	D7	42.2	-5.3	89.7	24.1	193.0	

Supplementary Table 32. Comparisons by sex for *Mmp-13* mRNA expression.

sex	diet	age	day	lsmean	lower.CL	upper.CL	SE	df	p.value
female	HFD	young	D3	214.6	171.3	258.0	22.0	193.0	<0.001
male	HFD	young	D3	88.3	40.8	135.8	24.1	193.0	
female	HFD	young	D7	228.5	181.0	276.0	24.1	193.0	<0.001
male	HFD	young	D7	41.0	-2.4	84.3	22.0	193.0	
female	LFD	young	D3	154.3	110.9	197.6	22.0	193.0	<0.001
male	LFD	young	D3	42.2	2.1	82.3	20.3	193.0	

Supplementary Table 33. Comparisons by age for *Mmp-13* mRNA expression.

age	diet	sex	day	lsmean	lower.CL	upper.CL	SE	df	p.value
old	HFD	female	D3	83.9	40.5	127.2	22.0	193.0	<0.001
young	HFD	female	D3	214.6	171.3	258.0	22.0	193.0	
old	HFD	female	D7	43.7	-9.4	96.8	26.9	193.0	<0.001
young	HFD	female	D7	228.5	181.0	276.0	24.1	193.0	
old	LFD	female	D3	42.1	-5.4	89.6	24.1	193.0	<0.001
young	LFD	female	D3	154.3	110.9	197.6	22.0	193.0	

Supplementary Table 34. Summary of coefficients of model for *Mmp-13* mRNA expression: age + sex + diet + day + age:sex + age:diet + sex:diet + age:day + sex:day + diet:day + age:sex:day + age:diet:day + sex:diet:day.

	Estimate	Std. Error	t value	Pr(>|t|)
(Intercept)	3.464	20.4	0.169	0.866
age - young	-4.597	26.6	-0.173	0.863
sex - male	-4.017	26.0	-0.155	0.877
diet - LFD	-3.861	26.6	-0.145	0.885
dayD3	78.814	29.0	2.718	0.007
dayD7	63.007	31.9	1.974	0.050
dayD14	7.473	29.0	0.258	0.797
dayD21	4.276	29.1	0.147	0.883
age - young:sex - male	8.778	30.2	0.291	0.771
age - young:diet - LFD	7.300	30.2	0.242	0.809
sex - male:diet - LFD	6.534	30.2	0.216	0.829
age - young:dayD3	138.544	37.9	3.653	0.000
age - young:dayD7	148.365	40.2	3.693	0.000
age - young:dayD14	3.324	38.6	0.086	0.931
age - young:dayD21	-1.659	39.6	-0.042	0.967
sex - male:dayD3	-11.268	37.5	-0.301	0.764

sex - male:dayD7	-54.900	39.2	-1.399	0.163
sex - male:dayD14	7.608	37.5	0.203	0.840
sex - male:dayD21	-2.421	38.3	-0.063	0.950
diet - LFD:dayD3	-34.428	38.5	-0.893	0.373
diet - LFD:dayD7	-43.419	39.7	-1.094	0.275
diet - LFD:dayD14	-4.227	38.0	-0.111	0.911
diet - LFD:dayD21	0.119	38.1	0.003	0.998
age - young:sex - male:dayD3	-123.338	43.6	-2.826	0.005
age - young:sex - male:dayD7	-103.912	44.5	-2.336	0.020
age - young:sex - male:dayD14	-12.133	43.8	-0.277	0.782
age - young:sex - male:dayD21	-1.981	44.2	-0.045	0.964
age - young:diet - LFD:dayD3	-32.576	43.6	-0.746	0.456
age - young:diet - LFD:dayD7	-109.833	44.4	-2.474	0.014
age - young:diet - LFD:dayD14	-5.003	43.8	-0.114	0.909
age - young:diet - LFD:dayD21	-2.948	44.2	-0.067	0.947
sex - male:diet - LFD:dayD3	14.236	43.6	0.326	0.745
sex - male:diet - LFD:dayD7	120.216	44.5	2.700	0.008
sex - male:diet - LFD:dayD14	-7.140	43.8	-0.163	0.871
sex - male:diet - LFD:dayD21	-2.235	44.2	-0.051	0.960

Statistically significant differences for *Tgfβ-1* mRNA expression by diet, sex and age

Supplementary Table 35. Comparisons by diet for *Tgfβ-1 mRNA expression.*

diet	age	sex	day	lsmean	lower.CL	upper.CL	SE	df	p.value
HFD	old	male	D0	6.6	3.5	9.7	1.6	199.0	0.038
LFD	old	male	D0	11.3	8.2	14.4	1.6	199.0	
HFD	young	female	D0	7.4	4.3	10.5	1.6	199.0	0.043
LFD	young	female	D0	11.9	8.8	15.0	1.6	199.0	

Supplementary Table 36. Comparisons by sex for *Tgfβ-1* mRNA expression.

sex	diet	age	day	lsmean	lower.CL	upper.CL	SE	df	p.value
female	HFD	old	D3	25.0	21.7	28.4	1.7	199.0	<0.001
male	HFD	old	D3	16.2	12.8	19.5	1.7	199.0	
female	HFD	old	D7	22.2	18.1	26.3	2.1	199.0	0.004
male	HFD	old	D7	14.4	11.0	17.7	1.7	199.0	
female	HFD	old	D14	14.2	10.8	17.5	1.7	199.0	0.016
male	HFD	old	D14	8.3	5.0	11.7	1.7	199.0	
female	LFD	old	D21	13.3	10.0	16.7	1.7	199.0	0.004
male	LFD	old	D21	6.3	2.9	9.7	1.7	199.0	

Supplementary Table 37. Comparisons by age for *Tgfβ-1* mRNA expression.

age	diet	sex	day	lsmean	lower.CL	upper.CL	SE	df	p.value
old	HFD	female	D3	25.0	21.7	28.4	1.7	199.0	<0.001
young	HFD	female	D3	13.8	10.5	17.2	1.7	199.0	
old	HFD	female	D7	22.2	18.1	26.3	2.1	199.0	0.014

young	HFD	female	D7	15.3	11.6	19.0	1.9	199.0	
old	HFD	female	D14	14.2	10.8	17.5	1.7	199.0	0.008
young	HFD	female	D14	7.7	4.3	11.0	1.7	199.0	
old	HFD	female	D21	11.4	8.0	14.7	1.7	199.0	0.020
young	HFD	female	D21	5.7	2.4	9.1	1.7	199.0	
old	LFD	female	D3	26.0	22.3	29.7	1.9	199.0	<0.001
young	LFD	female	D3	13.0	9.6	16.4	1.7	199.0	
old	LFD	female	D7	20.1	16.7	23.4	1.7	199.0	0.008
young	LFD	female	D7	13.3	9.6	17.0	1.9	199.0	

Supplementary Table 38. Summary of coefficients of model for *Tgfβ-1* mRNA expression: age + sex + diet + day + age:sex + age:day + sex:day + diet:day + age:sex:day.

	Estimate	Std. Error	t value	Pr(>|t|)
(Intercept)	7.272	1.20	6.053	0.000
age - young	0.522	1.56	0.335	0.738
sex - male	-0.204	1.56	-0.131	0.896
diet - LFD	3.717	1.12	3.308	0.001
dayD3	17.694	1.81	9.762	0.000
dayD7	13.821	1.92	7.186	0.000
dayD14	5.549	1.79	3.094	0.002
dayD21	4.233	1.79	2.360	0.019
age - young:sex - male	-0.470	2.25	-0.209	0.835
age - young:dayD3	-12.600	2.32	-5.429	0.000
age - young:dayD7	-7.168	2.42	-2.967	0.003
age - young:dayD14	-4.081	2.29	-1.780	0.076
age - young:dayD21	-4.718	2.29	-2.058	0.041
sex - male:dayD3	-8.583	2.32	-3.698	0.000
sex - male:dayD7	-5.267	2.36	-2.235	0.026
sex - male:dayD14	-4.462	2.29	-1.947	0.053
sex - male:dayD21	-5.828	2.32	-2.511	0.013
diet - LFD:dayD3	-2.637	1.65	-1.599	0.111
diet - LFD:dayD7	-3.983	1.68	-2.373	0.019
diet - LFD:dayD14	-3.682	1.65	-2.237	0.026
diet - LFD:dayD21	-2.044	1.65	-1.242	0.216
age - young:sex - male:dayD3	9.062	3.29	2.753	0.006
age - young:sex - male:dayD7	4.185	3.36	1.246	0.214
age - young:sex - male:dayD14	4.810	3.29	1.461	0.145
age - young:sex - male:dayD21	7.095	3.29	2.155	0.032

Statistically significant differences for *Tgfβ-3* mRNA expression by diet, sex and age

Supplementary Table 39. Comparisons by diet for *Tgfβ-3* mRNA expression.

diet	age	sex	day	lsmean	lower.CL	upper.CL	SE	df	p.value
HFD	old	female	D14	22.4	16.6	28.2	2.9	199.0	0.010
LFD	old	female	D14	11.6	5.8	17.4	2.9	199.0	

Supplementary Table 40. Comparisons by sex for *Tgfβ-3* mRNA expression.

sex	diet	age	day	lsmean	lower.CL	upper.CL	SE	df	p.value
female	HFD	old	D14	22.4	16.6	28.2	2.9	199.0	0.005
male	HFD	old	D14	10.5	4.7	16.3	2.9	199.0	

Supplementary Table 41. Comparisons by age for *Tgfβ-3* mRNA expression.

age	diet	sex	day	lsmean	lower.CL	upper.CL	SE	df	p.value
old	HFD	female	D14	22.4	16.6	28.2	2.9	199.0	0.003
young	HFD	female	D14	10.1	4.3	15.9	2.9	199.0	
old	HFD	male	D7	24.9	19.1	30.7	2.9	199.0	0.023
young	HFD	male	D7	15.4	9.6	21.2	2.9	199.0	

Supplementary Table 42. Summary of coefficients of model for *Tgfβ-3* mRNA expression: age + sex + diet + day + age:sex + age:diet + sex:diet + age:day + sex:day + age:sex:diet + age:sex:day.

	Estimate	Std. Error	t value	Pr(>|t|)
(Intercept)	10.098	2.10	4.806	0.000
age - young	0.361	3.01	0.120	0.905
sex - male	5.087	3.00	1.693	0.092
diet - LFD	-0.357	1.90	-0.188	0.851
dayD3	-2.401	2.92	-0.822	0.412
dayD7	9.642	3.02	3.194	0.002
dayD14	7.071	2.85	2.477	0.014
dayD21	2.655	2.85	0.930	0.353
age - young:sex - male	-3.564	4.35	-0.819	0.414
age - young:diet - LFD	3.079	2.67	1.155	0.249
sex - male:diet - LFD	3.288	2.66	1.238	0.217
age - young:dayD3	-1.757	4.08	-0.431	0.667
age - young:dayD7	-1.819	4.26	-0.427	0.669
age - young:dayD14	-6.468	4.04	-1.603	0.110
age - young:dayD21	-2.885	4.04	-0.715	0.475
sex - male:dayD3	-6.050	4.08	-1.482	0.140
sex - male:dayD7	-2.736	4.15	-0.659	0.511
sex - male:dayD14	-11.855	4.04	-2.938	0.004
sex - male:dayD21	-11.690	4.08	-2.862	0.005
age - young:sex - male:diet - LFD	-6.229	3.76	-1.656	0.099
age - young:sex - male:dayD3	8.675	5.79	1.497	0.136
age - young:sex - male:dayD7	-1.104	5.92	-0.187	0.852
age - young:sex - male:dayD14	14.318	5.79	2.471	0.014
age - young:sex - male:dayD21	11.601	5.79	2.002	0.047

Statistically significant differences for *Collagen 1* mRNA expression by diet, sex and age

Supplementary Table 43. Comparisons by diet for *Collagen 1* mRNA expression.

diet	age	sex	day	lsmean	lower.CL	upper.CL	SE	df	p.value
HFD	old	female	D14	10.5	5.9	15.0	2.3	199.0	0.050
LFD	old	female	D14	4.1	-0.5	8.6	2.3	199.0	
HFD	young	male	D21	28.1	23.5	32.6	2.3	199.0	0.019
LFD	young	male	D21	20.4	15.9	24.9	2.3	199.0	

Supplementary Table 44. Comparisons by sex for *Collagen 1* mRNA expression.

sex	diet	age	day	lsmean	lower.CL	upper.CL	SE	df	p.value
female	HFD	old	D0	1.5	-2.4	5.4	2.0	199.0	0.049
male	HFD	old	D0	7.3	3.1	11.4	2.1	199.0	
female	HFD	young	D0	2.4	-1.8	6.6	2.1	199.0	<0.001
male	HFD	young	D0	24.6	20.0	29.1	2.3	199.0	
female	HFD	young	D7	14.8	9.8	19.7	2.5	199.0	<0.001
male	HFD	young	D7	33.8	29.3	38.4	2.3	199.0	
female	HFD	young	D14	6.8	2.2	11.3	2.3	199.0	<0.001
male	HFD	young	D14	31.2	26.7	35.7	2.3	199.0	
female	HFD	young	D21	4.7	0.1	9.2	2.3	199.0	<0.001
male	HFD	young	D21	28.1	23.5	32.6	2.3	199.0	
female	LFD	young	D0	3.6	-0.6	7.8	2.1	199.0	<0.001
male	LFD	young	D0	25.5	21.0	30.1	2.3	199.0	
female	LFD	young	D3	4.2	-0.3	8.8	2.3	199.0	0.018
male	LFD	young	D3	11.7	7.5	15.9	2.1	199.0	
female	LFD	young	D7	7.8	2.8	12.8	2.5	199.0	<0.001
male	LFD	young	D7	31.2	26.7	35.7	2.3	199.0	
female	LFD	young	D14	6.4	1.9	11.0	2.3	199.0	<0.001
male	LFD	young	D14	28.1	23.2	33.1	2.5	199.0	
female	LFD	young	D21	5.2	0.7	9.7	2.3	199.0	<0.001
male	LFD	young	D21	20.4	15.9	24.9	2.3	199.0	

Supplementary Table 45. Comparisons by age for *Collagen 1* mRNA expression.

age	diet	sex	day	lsmean	lower.CL	upper.CL	SE	df	p.value
old	HFD	male	D0	7.3	3.1	11.4	2.1	199.0	<0.001
young	HFD	male	D0	24.6	20.0	29.1	2.3	199.0	
old	HFD	male	D3	4.5	0.0	9.1	2.3	199.0	0.049
young	HFD	male	D3	11.3	6.3	16.3	2.5	199.0	
old	HFD	male	D7	11.8	7.3	16.3	2.3	199.0	<0.001
young	HFD	male	D7	33.8	29.3	38.4	2.3	199.0	
old	HFD	male	D14	10.4	5.9	14.9	2.3	199.0	<0.001
young	HFD	male	D14	31.2	26.7	35.7	2.3	199.0	
old	HFD	male	D21	9.1	4.1	14.0	2.5	199.0	<0.001
young	HFD	male	D21	28.1	23.5	32.6	2.3	199.0	
old	LFD	male	D0	4.2	-0.0	8.4	2.1	199.0	<0.001
young	LFD	male	D0	25.5	21.0	30.1	2.3	199.0	

old	LFD	male	D3	5.4	0.8	9.9	2.3	199.0	0.045
young	LFD	male	D3	11.7	7.5	15.9	2.1	199.0	
old	LFD	male	D7	14.4	9.9	19.0	2.3	199.0	<0.001
young	LFD	male	D7	31.2	26.7	35.7	2.3	199.0	
old	LFD	male	D14	8.1	3.6	12.7	2.3	199.0	<0.001
young	LFD	male	D14	28.1	23.2	33.1	2.5	199.0	
old	LFD	male	D21	6.1	1.5	10.6	2.3	199.0	<0.001
young	LFD	male	D21	20.4	15.9	24.9	2.3	199.0	

Supplementary Table 46. Summary of coefficients of model for *Collagen 1* mRNA expression: age + sex + diet + day + age:sex + age:day + sex:day + age:sex:day.

	Estimate	Std. Error	t value	Pr(>|t|)
(Intercept)	2.521	1.519	1.659	0.098
age - young	1.220	2.104	0.580	0.563
sex - male	3.938	2.104	1.871	0.063
diet - LFD	-1.510	0.722	-2.092	0.038
dayD3	3.079	2.243	1.373	0.171
dayD7	7.845	2.308	3.399	0.001
dayD14	5.500	2.190	2.511	0.013
dayD21	3.424	2.190	1.563	0.119
age - young:sex - male	18.123	3.037	5.967	0.000
age - young:dayD3	-1.468	3.134	-0.468	0.640
age - young:dayD7	0.457	3.261	0.140	0.889
age - young:dayD14	-1.879	3.097	-0.607	0.545
age - young:dayD21	-1.479	3.097	-0.477	0.634
sex - male:dayD3	-3.826	3.134	-1.221	0.223
sex - male:dayD7	-0.431	3.181	-0.135	0.892
sex - male:dayD14	-1.925	3.097	-0.622	0.535
sex - male:dayD21	-1.630	3.134	-0.520	0.604
age - young:sex - male:dayD3	-11.187	4.448	-2.515	0.013
age - young:sex - male:dayD7	-0.400	4.538	-0.088	0.930
age - young:sex - male:dayD14	2.989	4.448	0.672	0.502
age - young:sex - male:dayD21	-1.122	4.448	-0.252	0.801

Statistically significant differences for *Collagen 3* mRNA expression by diet, sex and age

Supplementary Table 47. Comparisons by diet for *Collagen 3* mRNA expression.

Diet	age	sex	day	lsmean	lower.CL	upper.CL	SE	df	p.value
HFD	young	female	D7	22.8	16.5	29.0	3.2	199.0	0.021
LFD	young	female	D7	12.3	6.1	18.6	3.2	199.0	

Supplementary Table 48. Comparisons by sex for *Collagen 3* Mrna expression.

sex	diet	age	day	lsmean	lower.CL	upper.CL	SE	df	p.value
female	HFD	young	D0	2.4	-2.9	7.7	2.7	199.0	<0.001
male	HFD	young	D0	24.5	18.8	30.2	2.9	199.0	
female	HFD	young	D3	9.9	4.1	15.6	2.9	199.0	<0.001
male	HFD	young	D3	24.7	18.4	30.9	3.2	199.0	
female	HFD	young	D7	22.8	16.5	29.0	3.2	199.0	<0.001
male	HFD	young	D7	52.1	46.3	57.8	2.9	199.0	
female	HFD	young	D14	9.0	3.2	14.7	2.9	199.0	<0.001
male	HFD	young	D14	42.5	36.8	48.3	2.9	199.0	
female	HFD	young	D21	3.6	-2.1	9.3	2.9	199.0	<0.001
male	HFD	young	D21	31.5	25.8	37.2	2.9	199.0	
female	LFD	young	D0	3.2	-2.1	8.5	2.7	199.0	<0.001
male	LFD	young	D0	26.6	20.8	32.3	2.9	199.0	
female	LFD	young	D3	7.4	1.6	13.1	2.9	199.0	<0.001
male	LFD	young	D3	21.8	16.5	27.2	2.7	199.0	
female	LFD	young	D7	12.3	6.1	18.6	3.2	199.0	<0.001
male	LFD	young	D7	53.5	47.8	59.3	2.9	199.0	
female	LFD	young	D14	7.8	2.1	13.6	2.9	199.0	<0.001
male	LFD	young	D14	38.3	32.0	44.6	3.2	199.0	
female	LFD	young	D21	5.7	-0.0	11.4	2.9	199.0	<0.001
male	LFD	young	D21	28.3	22.6	34.0	2.9	199.0	

Supplementary Table 49. Comparisons by age for *Collagen 3* mRNA expression.

age	diet	sex	day	lsmean	lower.CL	upper.CL	SE	df	p.value
old	HFD	female	D7	6.0	-1.0	13.0	3.6	199.0	<0.001
young	HFD	female	D7	22.8	16.5	29.0	3.2	199.0	
old	HFD	male	D0	1.9	-3.4	7.2	2.7	199.0	<0.001
young	HFD	male	D0	24.5	18.8	30.2	2.9	199.0	
old	HFD	male	D3	6.4	0.7	12.2	2.9	199.0	<0.001
young	HFD	male	D3	24.7	18.4	30.9	3.2	199.0	
old	HFD	male	D7	6.6	0.9	12.3	2.9	199.0	<0.001
young	HFD	male	D7	52.1	46.3	57.8	2.9	199.0	
old	HFD	male	D14	7.3	1.5	13.0	2.9	199.0	<0.001
young	HFD	male	D14	42.5	36.8	48.3	2.9	199.0	
old	HFD	male	D21	4.2	-2.1	10.5	3.2	199.0	<0.001
young	HFD	male	D21	31.5	25.8	37.2	2.9	199.0	
old	LFD	male	D0	1.0	-4.3	6.3	2.7	199.0	<0.001
young	LFD	male	D0	26.6	20.8	32.3	2.9	199.0	
old	LFD	male	D3	7.4	1.7	13.1	2.9	199.0	<0.001
young	LFD	male	D3	21.8	16.5	27.2	2.7	199.0	
old	LFD	male	D7	11.9	6.1	17.6	2.9	199.0	<0.001
young	LFD	male	D7	53.5	47.8	59.3	2.9	199.0	
old	LFD	male	D14	5.7	-0.0	11.4	2.9	199.0	<0.001
young	LFD	male	D14	38.3	32.0	44.6	3.2	199.0	
old	LFD	male	D21	3.4	-2.3	9.1	2.9	199.0	<0.001
young	LFD	male	D21	28.3	22.6	34.0	2.9	199.0	

Supplementary Table 50. Summary of coefficients of model for *Collagen 3* mRNA expression: age + sex + day + age:sex + age:day + sex:day + age:sex:day.

	Estimate	Std. Error	t value	Pr(>|t|)
(Intercept)	0.575	1.86	0.309	0.758
age - young	2.195	2.63	0.833	0.406
sex - male	0.880	2.63	0.334	0.739
dayD3	4.279	2.81	1.524	0.129
dayD7	4.287	2.88	1.486	0.139
dayD14	2.119	2.74	0.773	0.440
dayD21	1.766	2.74	0.644	0.520
age - young:sex - male	21.874	3.80	5.753	0.000
age - young:dayD3	1.558	3.92	0.397	0.692
age - young:dayD7	10.489	4.08	2.570	0.011
age - young:dayD14	3.514	3.88	0.906	0.366
age - young:dayD21	0.099	3.88	0.026	0.980
sex - male:dayD3	1.199	3.92	0.305	0.760
sex - male:dayD7	3.485	3.98	0.876	0.382
sex - male:dayD14	2.920	3.88	0.753	0.452
sex - male:dayD21	0.557	3.92	0.142	0.887
age - young:sex - male:dayD3	-9.533	5.57	-1.712	0.088
age - young:sex - male:dayD7	9.017	5.68	1.587	0.114
age - young:sex - male:dayD14	6.525	5.57	1.172	0.243
age - young:sex - male:dayD21	1.953	5.57	0.351	0.726

Statistically significant differences for hydroxyproline content by diet and age

Supplementary Table 51. Summary of coefficients of model for hydroxyproline content: age + sex + diet + day + age:diet + age:day + diet:day + age:diet:day.

	Estimate	Std. Error	t value	Pr(>|t|)
(Intercept)	3.967	0.570	6.963	0.000
age- young	1.031	0.806	1.279	0.205
diet- LFD	0.577	0.806	0.716	0.476
dayD14	-0.655	0.834	-0.785	0.435
dayD21	-0.268	0.987	-0.271	0.787
age- young:diet- LFD	-1.602	1.160	-1.382	0.171
age- young:dayD14	-0.440	1.160	-0.380	0.705
age -young:dayD21	-2.563	1.274	-2.012	0.048
diet -LFD:dayD14	1.030	1.160	0.888	0.377
diet- LFD:dayD21	-1.117	1.292	-0.864	0.390
age- young:dietLFD:dayD14	-0.076	1.640	-0.046	0.963
age- young:dietLFD:dayD21	3.607	1.736	2.078	0.041

Statistically significant differences for fibrosis by diet and age

Supplementary Table 52. Comparisons by age for fibrosis.

age	diet	day	lsmean	lower.CL	upper.CL	SE	df	p.value
old	LFD	D14	18.3	12.7	23.9	2.7	16.0	0.028
young	LFD	D14	27.4	21.8	33.0	2.7	16.0	
old	LFD	D21	13.8	8.1	19.4	2.7	16.0	<0.001
young	LFD	D21	29.2	23.6	34.9	2.7	16.0	

Supplementary Table 53. Summary of coefficients of model for fibrosis: age * diet * day.

	Estimate	Std. Error	t value	Pr(>|t|)
(Intercept)	21.54	2.66	8.111	0.000
age - young	7.36	3.76	1.961	0.068
diet - LFD	-3.23	3.76	-0.860	0.402
dayD21	-2.19	3.76	-0.583	0.568
age - young:diet - LFD	1.73	5.31	0.326	0.749
age - young:dayD21	-5.10	5.31	-0.960	0.351
diet - LFD:dayD21	-2.36	5.31	-0.444	0.663
age - young:diet - LFD:dayD21	11.49	7.51	1.529	0.146
